# A Mini Review on Isatin, an Anticancer Scaffold with Potential Activities against Neglected Tropical Diseases (NTDs)

**DOI:** 10.3390/ph15050536

**Published:** 2022-04-27

**Authors:** Shefali Chowdhary, Shalini, Amandeep Arora, Vipan Kumar

**Affiliations:** 1 Department of Chemistry, Guru Nanak Dev University, Amritsar 143005, India; shefalich39@gmail.com (S.C.); shalini.bhanot27@gmail.com (S.); 2 Department of Natural and Applied Sciences, University of Dubuque, Dubuque, IA 52002, USA; aarora@dbq.edu

**Keywords:** isatin, antiproliferatives, antiplasmodials, antimycobacterials, antimicrobials

## Abstract

Isatin, chemically an indole-1*H*-2,3-dione, is recognised as one of the most attractive therapeutic fragments in drug design and development. The template has turned out to be exceptionally useful for developing new anticancer scaffolds, as evidenced by the increasing number of isatin-based molecules which are either in clinical use or in trials. Apart from its promising antiproliferative properties, isatin has shown potential in treating Neglected Tropical Diseases (NTDs) not only as a parent core, but also by attenuating the activities of various pharmacophores. The objective of this mini-review is to keep readers up to date on the latest developments in the biological potential of isatin-based scaffolds, targeting cancer and NTDs such as tuberculosis, malaria, and microbial infections.

## 1. Introduction

Neglected Tropical Diseases cause health, social, and economic problems in tropical and subtropical regions and affect the world’s poorest and most vulnerable people. They are most common in the tropical and subtropical areas of Africa, Asia, and Latin America, where hot and humid climatic conditions promote vector growth [1]. These are commonly referred to as NTDs because they receive little attention in terms of surveillance, prevention, and treatment. Poor sanitation and hygiene, the development of resistant strains, and the toxicity of current antiprotozoal regimens are the primary causes of the rise in the global burden of protozoan diseases. According to WHO statistics, this broad group of ailments affects more than one billion people worldwide and is found in 149 tropical and subtropical countries. NTDs restrict afflicted communities severely, resulting in a slew of illnesses, pain, disability, and death, as well as significant social, economic, and psychological implications for millions of people [2]. Initiatives from all over the world have been critical in the fight against NTDs. The Drugs for Neglected Diseases Initiative (DNDi) is a non-profit research organisation dedicated to the development of novel medications for people suffering from neglected diseases [3]. Aside from de novo drug discovery, the repositioning and re-engineering of existing drugs or drug-like molecules with known pharmacokinetics and established target profiles are excellent starting points for identifying new chemical entities. 

Isatin, also known as 1*H*-indol-2,3-dione, is a natural heterocyclic compound extracted as red-orange powder from a variety of plants worldwide, including *Isatis tinctoria*, *Couroupita guianensis Aubl*, *Melochia tomentosa*, and *Boronia koniamboensis* [4]. Moreover, it is also found in the secretion of the parotid gland of Bufo frogs as well as in the Australian mollusc *Dicathais orbita* [5]. In humans, it is identified as a metabolite of tryptophan or epinephrine and is largely distributed in the central nervous system (CNS), peripheral tissues, as well as body fluids. Substituted isatins can also be found in plants such as melosatin alkaloids (methoxy phenyl isatins) isolated from the Caribbean tumourigenic plant *Melochia tomentosa*, as well as in fungi, such as 6-(3-Methylbuten-2’-yl) isatin and 5-(3’-Methylbuten-2’-yl) isatin isolated from *Streptomyces albus* [6]. Among the most important biological activities of isatin derivatives are anticancer, antitubercular, antimalarial, antifungal, antibacterial, anticonvulsant, and antiviral properties. Despite the dominance of biological applications, other areas such as catalysis, dye compounds, nanocomposites, and polymers have merited special attention. Isatin derivatives possess a wide range of applications due to their inherent versatility in structure, which allows for the construction of diverse frameworks suitable for a specific biological or chemical property of interest. A variety of substituents, particularly at N-1, C-3 (C=O) and C5/C6/C7 (aromatic), can be introduced, modulating both the biological and chemical properties of the parent core [7].

Although a number of reviews on the synthesis and biological applications of isatins have recently appeared [8,9], the current review’s goal is to keep readers up to date on the biological applications of isatin derivatives and isatin hybrids, with a focus on cancer and Neglected Tropical Diseases such as malaria, tuberculosis, bacterial infections, and parasitic diseases. 

## 2. Anticancer Activities of Functionalized Isatins

In every country on this planet, cancer is the main source of mortality and a key hindrance to extending life expectancy. Cancer is the primary or second largest cause of death among people under the age of seventy, according to the World Health Organization (WHO). Globally, 19.3 million new cancer cases are expected to be diagnosed in 2020, with about 10.0 million cancer deaths [10]. Female breast cancer has superseded lung cancer as the most frequently diagnosed cancer, with an estimated 2.3 million new cases, followed by lung, colorectal, prostate, and stomach cancers. The unprecedented diversity of cancer continues to provide insights to the underlying factors, but it also perpetuates the need for a global intensification of measures to control the disease [11].

**Figure 1:**
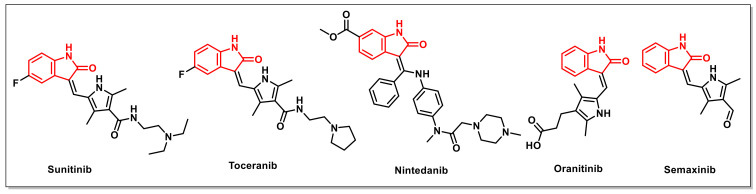
Structures of isatin derivatives approved as drugs or in clinical trials.

A number of isatin-based compounds have entered into clinical trials, including two compounds, namely Sunitinib and Toceranib, that have been approved for clinical use against tumours (Figure 1). Sunitinib suppresses the catalytic activity of kinases in the phosphorylation of proteins by reversibly binding to their ATP binding sites. Toceranib, a Sunitinib-like molecule, acts as a selective inhibitor of specific receptor tyrosine kinases (RTKs), thus triggering tumour cells’ apoptosis in vivo. Other derivatives, including Nintedanib, Semaxinib, and Orantinib, are currently undergoing clinical trials for their anticancer potential (Figure 1). These promising molecules have demonstrated the capacity to slow or stop the growth of tumours via modulating cell growth, proliferation, survival, and migration. However, some of these anticancer candidates also display side effects, such as limited efficacy, diarrhoea, hypertension, vomiting, hand–foot syndrome, and neutropenia, which provides an impetus for the identification of new candidates with promising activities [12].

### 2.1. Isatin-Based Amides and Sulphonamides

**Figure 2:**
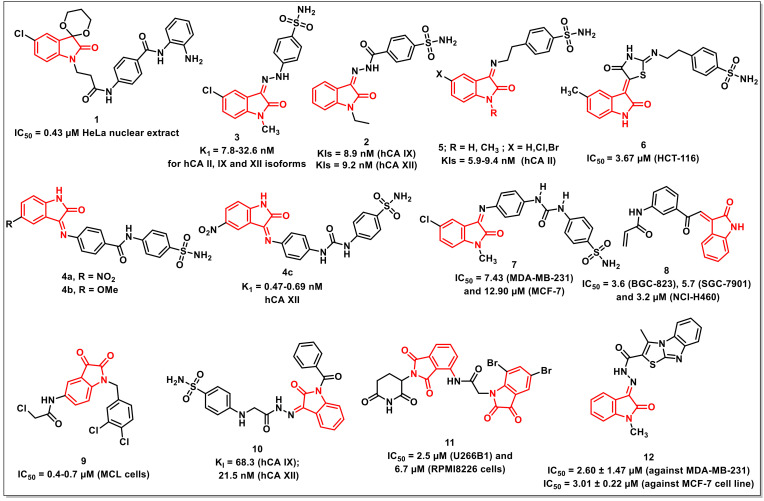
Amide- and sulphonamide-based isatin scaffolds with promising antiproliferative/anticancer activities.

Gao et al. synthesized a series of compounds, having isatin as caps and *o*-phenylenediamine as a zinc-binding functionality. The synthesized scaffolds were evaluated for their HDAC inhibition using HeLa nuclear extract. Compound **1** proved to be the most active among the series, with comparable HDAC inhibition and antiproliferative activities with entinostat (MS-275) (Figure 2). However, **1** exhibited moderate HDAC1 selectivity over HDAC2 and HDAC3, as compared to entinostat (MS-275) [13]. 

Abo-Ashour et al. synthesized a series of hydrazide-linked isatin-sulfonamides and evaluated various carbonic anhydrase isoforms *viz.* hCA I, II, IX, and XII. The synthesized scaffolds inhibited the tested hCAs to varying degrees; for example, the KIs on hCA I ranged from 671.8 to 3549.5 nM, while, for hCA II, hCA IX, and hCA XII, it ranged from 36.8 to 892.4; 8.9 to 264.5, and 9.0 to 78.1 nM, respectively. The isatin-sulfonamide **2** proved to be the most active among the synthesized series, exhibiting KIs of 8.9 and 9.2 nM on hCA IX and XII, respectively (Figure 2). Molecular docking studies of **2** within the hCA II, IX, and XII active sites were also performed in order to rationalize the observed results [14]. 

The authors further extended the study and synthesized a series of isatin-benzenesulfonamide hybrids linked via a hydrazine linker and evaluated them against tumour-associated human carbonic anhydrase isoforms, hCA I, II, IX, and XII, along with their antiproliferative evaluation on a panel of sixty cancer lines according to US-NCI protocol. The synthesized compounds exhibited inhibitory activities with K_1_ ranging from 28.3 to 692.2; 8.3 to 65.4; and 11.9 to 72.9 nM on hCA II, IX, and XII isoforms, respectively. The promising compound among the synthesized series, **3** (K_1_ from 7.8–32.6 nM), exhibited broad spectrum activity on various cancer lines (Figure 2). Molecular modelling studies were also carried out in order to ascertain the possible binding interactions of the promising scaffolds in the active sites of isoforms II and IX [15]. 

Eldehna et al. synthesized a series of amido/ureido-tethered isatin-benzene sulfonamide hybrids and evaluated the in vitro inhibitory activity against a panel of hCA I and II (cytosolic) and IX and XII (transmembrane, tumour-associated) isoforms. Most of the synthesized hybrids inhibited the tested isoforms in variable degrees, with the best activity being observed on tumour-associated isoform hCA XII, with K_1_ values ranging from 0.47 to 2.83 nM. Among the amide-linked hybrids, the presence of either an electron-withdrawing (NO_2_) or electron-releasing (OCH_3_) substituent improved the activity profiles, as evidenced by hybrids **4a** and **4b** exhibiting Ki (inhibition constant) values of 0.69 and 0.47 nM, respectively (Figure 2). Among urea-linked hybrids, compound **4c**, with −NO_2_ substituent at the C-5 position of the isatin ring, proved to be promising, with a K_i_ value of 0.64 nM [16].

Further, a series of sulfonamide incorporating substituted indolin-2-ones through aminoethyl or (4-oxothiazolidin-2-ylidene) aminoethyl linkers was synthesized and evaluated in vitro on human carbonic anhydrase isoforms hCA I, II, IV, and VII. The synthesized scaffolds inhibited the tested hCAs in variable degrees. In the case of hCA I, the scaffolds exhibited Ki’s ranging from 42 to 8550.9 nM, while the Ki’s in case of hCA II, hCA IV, and hCA VII were in the range of 5.9–761; 4.0–2069.5; and 13.2–694 nM, respectively. The scaffold **5** proved to be most the promising hCA II inhibitors, with Ki’s in the range of 5.9 to 9.4 nM (Figure 2) [17].

In another report, Eldehna et al. disclosed the synthesis and in vitro evaluation of a series of indolinone-based sulfonamides as inhibitors of carbonic anhydrase isoforms, namely hCA IX and XII. The synthesized sulfonamides exhibited inhibitory activities in low nM concentration towards both hCA IX (Ki’s: 6.2–64.8 nM) and XII (Ki’s: 7.1–55.6 nM) isoforms. Further, the synthesized scaffolds were also evaluated for in vitro antiproliferative activities on HCT-116 and MCF-7 cells. Compound **6** proved to be most active on HCT-116, with an IC_50_ of 3.67 μM, and was further assayed for cell cycle disturbance and apoptosis induction in HCT-116 cells (Figure 2). The compound exhibited cell cycle arrest at the G2-M stage, along with an alteration of the Sub-G1 phase and induced intrinsic apoptotic mitochondrial pathway through down-regulation of the antiapoptotic protein Bcl-2 level [18]. 

The above authors further reported the synthesis of *N*-substituted isatin-SLC-0111 hybrids along with their evaluation on physiologically relevant hCA isoforms viz. hCA I, II, IX, and XII using a stopped flow CO_2_ hydrase assay. The synthesized hybrids efficiently inhibited hCA IX and XII, with Ki’s ranging from 4.7 to 86.1 nM and 1.3 to 80.9 nM, respectively. The promising hybrids were then evaluated for their antiproliferative activities on MDA-MB-231 and MCF-7 cells under hypoxic conditions and identified **7** as the most active hybrid with IC_50_s of 7.43 and 12.90 μM (Figure 2). Further, **7** disrupted the MDA-MB-231 cell cycle through the modification of the sub-G1 phase and arrested the G2-M phase. Additionally, **7** exhibited a substantial increase in annexinV-FITC positive apoptotic cells from 1.03 to 18.54%, along with potent VEGFR-2 inhibitory activity, with an IC_50_ of 260.64 nM [19]. 

Wang et al. synthesized a series of isatin-inspired *α*,*β*-unsaturated ketones and evaluated their antiproliferative activities on a panel of cancer cell lines using an MTT [(3-(4,5-dimethylthiazol-2-yl)-2,5-diphenyltetrazolium bromide)] assay. The initial screening revealed **8** as active on all the cell lines tested, being most active on BGC-823, SGC-7901 and NCI-H460 cells with IC_50_s of 3.6, 5.7, and 3.2 μM, respectively (Figure 2). Compound **8** effectively inhibited the growth of NCI-H460 cells in a concentrated and time-dependent manner, with cell cycle arrest in G2/M phase. Additionally, **8** inhibited the growth of an NCI-H460 xenograft tumour in vivo [20]. 

Yu et al. synthesized eighteen indoline-2,3-diones and evaluated their antiproliferative activities on mantle cell lymphoma (MCL) cells. The synthesized compounds displayed good antiproliferative activity, with compound **9** being most potent, with an IC_50_ of 0.4–0.7 μM on MCL cells (Figure 2). The compound induced cell apoptosis and cell cycle arrest in the G2/M phase in a dose-dependent manner [21]. 

George et al. disclosed the synthesis of isatin-benzenesulfonamides for assessing their inhibitory activities on human CA isoforms viz. hCA I, hCA II, hCA IX, and hCA XII. Most of the synthesized scaffolds displayed substantial inhibitory activities on tumour-associated isoforms IX and XII. Compound **10** revealed the highest activity among the synthesized scaffolds, with a K_i_ of 68.3 and 21.5 nM on hCA IX and hCA XII, respectively (Figure 2). Docking studies was carried out in order to understand the interactions between **10** and the four isoforms studied. As expected, **10** exhibited stronger interactions with isoform XII, while few interactions were observed with isoform IX. Further, the poor interactions of **10** with isoforms I and II confirmed its poor activity profiles [22]. 

Panga et al. synthesized a series of isatin-pomalidomide hybrids and assayed for in vitro antiproliferative activities against U266B1 and RPMI 8226 on multiple myeloma cells using an MTT assay with pomalidomide as the reference drug. Most of the synthesized hybrids exhibited moderate to good cytotoxicity on the tested cell lines. Among these, compound **11** exhibited an IC_50_ of 2.5 μM on U266B1 and 6.7 μM on RPMI8226 cells, which are values better than that of the physical mixture of 5,7-di-bromoisatin and pomalidomide (PM + DBIS) (Figure 2) [23]. 

Eldehna et al. aimed at developing a new set of small molecules featuring the isatin framework tethered with a thiazolo [3,2-*a*]benzimidazole (TBI) motif via a cleavable hydrazide linker as potential anticancer CDK2 inhibitors. Within the CDK2 binding region, the vast tricyclic TBI motif is expected to form plenty of hydrophobic contacts. Most of the synthesized hybrids substantially hindered the development of the two cell lines under investigation. Notably, **12** was the most potent moiety among these, with IC_50_ = 2.60 ± 1.47 μM against MDA-MB-231 and IC_50_ = 3.01 ± 0.22 μM against the MCF-7 cell line (Figure 2). The TBI ring’s capacity to well accommodate and form extensive hydrophobic interactions within a hydrophobic pocket in the CDK2 binding site was shown by docking simulations, as predicted. In addition, docking modelling emphasized the importance of hydrazide linkage and isatin unsubstituted (NH) functionality in H-bonding interactions [24].

### 2.2. 1H-1,2,3-Triazole-Tethered Isatin Hybrids

**Figure 3:**
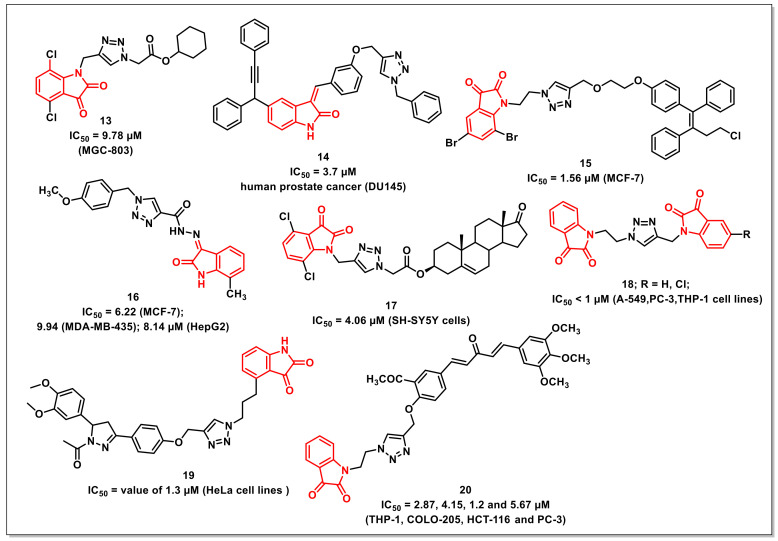
1*H*-1,2,3-triazole-based isatin compounds with antiproliferative potential.

Yu et al. synthesized a series of isatin-triazole hybrids and evaluated their antiproliferative activities against a panel of cancer cell lines. The synthesized hybrids exhibited moderate to good antiproliferative activities on tested cells, with selectivity against MGC-803. The most promising scaffold of the synthesized library, **13**, displayed an IC_50_ of 9.78 μM (MGC-803) and was less cytotoxic on normal cell lines viz. HL-7702 and GES-1, with IC_50_s of 40.27 and 35.97 μM, respectively (Figure 3). The hybrid **13** instigated morphological changes on the tested cell lines along with the induction of cell cycle arrest (G2/M phase), the generation of cellular ROS, and the inhibition of the migration of cells in a concentration-dependent manner [25]. 

Nagarsenkar et al. reported the synthesis and antiproliferative evaluation of a series of triazole-tethered 3-benzylidene-isatin hybrids. The synthesized hybrids were assessed for their cytotoxic potential on DU145 (prostate), PC-3 (prostate), MDA-MB-231 (breast), BT549 (breast), A549 (lung), and HeLa (cervical) human cancer cell lines using MTT assay. Among the synthesized series, compound **14** proved to be most potent, with an IC_50_ of 3.7 μM against DU145 cells (Figure 3). Its safety profile on normal prostate cells (RWPE-1) confirmed it to be non-cytotoxic. Compound **14** induced apoptosis on DU145 cells, as confirmed by AO/EB staining and Annexin V binding assay and DAPI nuclear staining with a cell cycle arrest at G2/M phase. The compound additionally resulted in the collapse of mitochondrial membrane potential and increased intracellular ROS levels in DU145 cells [26].

Kumar et al. reported a series of triazole-linked ospemifene-isatins and ospemifene-spiroisatins along with their antiproliferative evaluation on MCF-7 and MDA-MB-231 cells. SAR studies disclosed that the hybrids with bromo-substituent at the C-5 and C-7 positions of the isatin core, along with an ethyl/propyl as a spacer, were promising on MCF-7 cells. The compound **15** proved to be the most promising, with an IC_50_ value of 1.56 μM against MCF-7 (Figure 3) [27]. 

Aneja et al. reported the synthesis of isatin-triazole hydrazones as potent inhibitors of microtubule affinity-regulating kinase 4 (MARK4) on the basis of a toxicity analysis on MCF-7, HepG2, and MDA-MB-435s cells. Among the synthesized hydrazones, the compound **16** exhibited good binding affinity along with easy transportation, as confirmed by a Human Serum Albumin (HSA) binding assay (Figure 3). The compound induced apoptosis on MCF-7, MDA-MB-435s, and HepG2 cells with IC_50_s of 6.22, 9.94, and 8.14 μM, respectively. Additionally, the cell-based assay validated the cell promotion, apoptosis, and enhanced ROS generation by **16 [28]**. 

Yu et al. synthesized a series of 1*H*-1,2,3-triazole-tethered isatin-steroidal hybrids and evaluated their antiproliferative activities on a panel of different cancer cell lines using an MTT assay. The preliminary evaluation results indicated that the hybrids with a terminal isatin motif exhibited substantial growth inhibition of SH-SY5Y cells. Among the synthesized series, compound **17** proved to be the most active, and exhibited an IC_50_ of 4.06 μM against SH-SY5Y cells, comparable to that of 5-FU (Figure 3). Additionally, **17** arrested the cell cycle growth at the G2/M phase along with induction of apoptosis and subsequent lowering of mitochondrial membrane potential. The observed activity of **17** was further substantiated using Docking studies [29]. 

Singh et al. synthesized 1*H*-1,2,3-triazole-tethered isatin conjugates and evaluated for cytotoxicity on four human cancer cell lines. The results showed that **18** was twice as effective as 5-fluorouracil on the THP-1 cell line and had an IC_50_ of <1 μM against all cell lines except Caco-2 (Figure 3). Activity profiles showed a reliance on the substituents on isatin rings, with preference for hydrogen, while an electron-withdrawing fluoro and nitro substituents on either ring diminished the antiproliferative activity [30].

Sharma et al. synthesized 3,5-diaryl-*N*-acetyl-pyrazolines(DNAP)-isatin hybrids via Cu-promoted click reaction. The synthesized hybrids were assayed for their antiproliferative activity on four human cancer cell lines using a sulforhodamine B assay. The most potent hybrid **19** displayed an IC_50_ value of 1.3 μM (HeLa cells) with ten-fold selectivity towards HeLa cell lines (Figure 3). SAR studies revealed that both the electronic effects and the length of the linker remarkably affected the cytotoxic activity of the synthesized hybrids. The inclusion of a propyl chain as a spacer and methoxy substituents on the phenyl ring of *N*-acetyl pyrazoline were considered optimum for the good antiproliferative activities of the synthesized hybrids [31]. 

Further, a series of 1*H*-1,2,3-triazole-tethered curcumin-isatin hybrids were synthesized by Sharma et al. and assayed for their cytotoxicity against THP-1, COLO-205, HCT-116, A549, HeLa, CAKI-I, PC-3, and MiaPaca-2 human cancer cells. The most potent compounds were also assayed for tubulin inhibition. Compound **20** was found to substantially inhibit the tubulin polymerization (IC_50_ = 2.87, 4.15, 1.2, and 5.67 μM against THP-1, COLO-205, HCT-116, and PC-3, respectively) (Figure 3). Additionally, **20** led to the disruption of microtubules, as affirmed by immunofluorescence studies. The docking study confirmed that **20** fits well at the interface of β1 and α2 subunits of tubulin and stabilized by H-bonds, polar, and van der Walls interactions [32].

### 2.3. Spiro Compounds Based on Isatins

**Figure 4:**
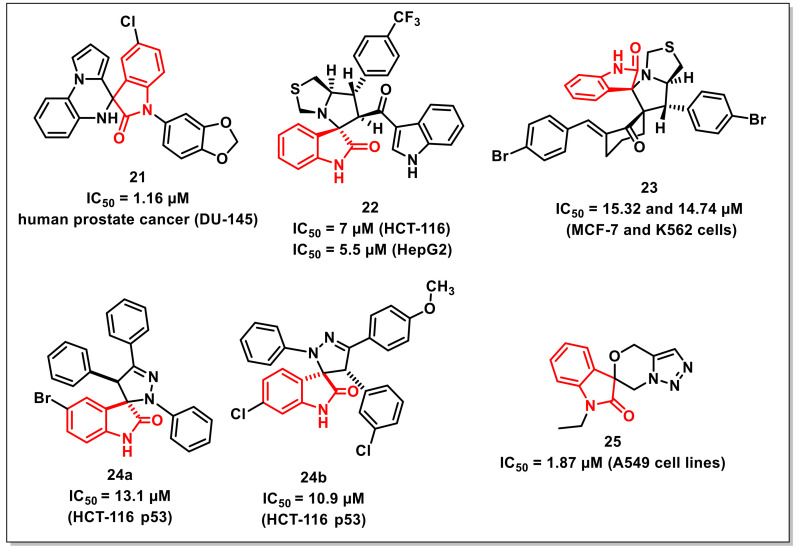
Spiro-isatins as antiproliferative scaffolds.

Kamal et al. synthesized and evaluated a series of 5′*H*-spiro[indoline-3,4′-pyrrolo [1,2-a]quinoxalin]-2-ones against a panel of cancer cell lines, taking doxorubicin as the positive control. The synthesized library exhibited moderate to good antiproliferative activities with IC_50_s ranging from 1.16 to 31.44 μM. The most promising compound of the series, **21**, with the presence of a piperonyl substituent, displayed significant activity against human prostate cancer (DU-145), with an IC_50_ of 1.16 μM (Figure 4). The scaffold induced cell cycle arrest in the G0/G1 phase along with a reduction in Cdk4 expression level, as indicated by flow cytometric and Western blot analysis, respectively. Further studies such as cell cycle analysis, mitochondrial potential assay, annexin V-FITC assay, and Western blot analysis of **21** confirmed apoptosis as its possible mechanism of action [33].

Islam et al. disclosed the synthesis and in vitro antiproliferative activities of a series of spirooxindole linked 3-acylindoles on hepatocellular carcinoma, colon, and prostate cancer cells. Among the synthesized scaffolds, compound **22** exhibited high cytotoxicity and selectivity against colon cancer cells, including HCT-116 with an IC_50_ of 7 μM (SI: 3.7) and HepG2 with an IC_50_ of 5.5 μM (SI: 4.7) (Figure 4). The antiproliferative studies of **22** on prostate cancer cells revealed that, although the compound was less active than the standard drug cisplatin, it still showed greater selectivity. The binding modes of the promising compounds were confirmed via molecular docking studies [34]. 

Lotfy et al. synthesized a series of spirooxindole-pyrrolidines through 1,3-dipolar cycloaddition reactions of azomethine ylides generated from isatins with alkenes. The synthesized scaffolds were evaluated for their in vitro antiproliferative activities against MCF-7 and K562 cells. The most promising compound among the series, **23**, exhibited IC_50_s of 15.32 and 14.74 μM against MCF-7 and K562 cells, respectively (Figure 4) [35]. 

Nunes et al. disclosed the synthesis and antiproliferative activities of a series of spiropyrazoline oxindoles on the HCT-116 p53 human colon cancer cell line. Out of the synthesized library, eight derivatives exhibited good activities with IC_50_s less than 15 μM. Among these potent compounds, two derivatives viz. **24a** (IC_50_ 13.1 μM) and **24b** (IC_50_ 10.9 μM) were selected to delineate the mechanism of action (Figure 4). Both scaffolds induced apoptosis with cell arrest at the G0/G1 phase along with up-regulation of p53 steady state levels. Further, the combination of **24a** with 5-fluorouracil (5-FU) fashioned a synergistic inhibitory effect on the proliferation of colon cancer cells [36]. 

Senwar et al. developed a concise method for the synthesis of spirooxindole-derived morpholine-fused-1,2,3-triazoles. The methodology included the regiospecific ring-opening of isatin-spiro-epoxides by azide as a nucleophile, followed by successive *O*-propargylation and intramolecular 1,3-dipolar cycloaddition. The synthesized compounds were assessed for their antiproliferative activities on various human cancer cell lines. Among these, compound **25** (IC_50_ = 1.87 μM) showed good growth inhibition on A549 cells (Figure 4). Flow cytometry investigation demonstrated that compound **25** arrested the cells at the G2/M period of the cell cycle. The apoptosis-inducing effect of these compounds was examined with Hoechst nuclear staining and AO/EB staining. Additionally, compound **25** prompted apoptosis in A549 cells through the breakdown of mitochondrial membrane potential and the elevation of intracellular responsive oxygen species levels [37].

### 2.4. Urea/Thiourea-Based Isatin-Derivatives

**Figure 5:**
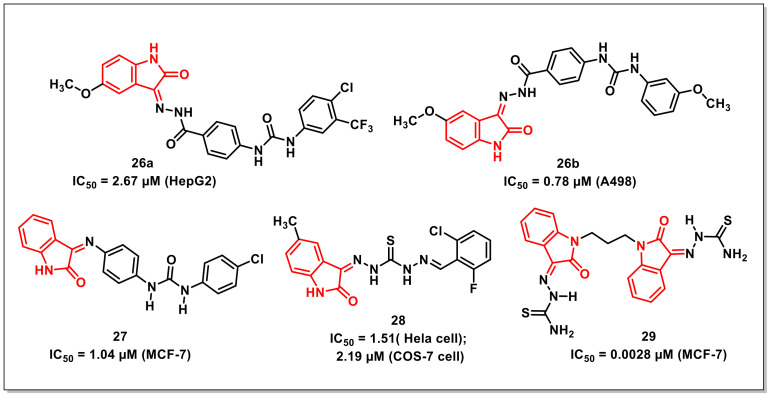
Urea/thiourea-based isatin compounds with anticancer potential.

Eldehna et al. disclosed the synthesis of a series of amide/urea-based indoline-2-ones via conjugating type IIA (sorafenib) and IIB (sunitinib and nintedanib) as PTK inhibitors. The synthesized hybrids were evaluated for multi-kinase inhibitory activity on VEGFR2, PDGFR-b and FGFR-1 and for antiproliferative activity on HepG2, MCF-7, A549, and A498 cancer cells. The hybrid **26a** proved to be the most potent multi-kinase inhibitor among the synthesized ureido series, with IC_50_s of 0.18, 0.23, and 0.10 μM on VEGFR-2, FGFR-1, and PDGFR-b, respectively (Figure 5). For antiproliferative activities, **26a** proved to be most potent on HepG2 cells (IC_50_ = 2.67 ± 0.14 μM), while **26b** was most potent on A498 cells (IC_50_ = 0.78 ± 0.02 μM). Further, the synthesized compounds proved to be non-cytotoxic over human normal melanocyte (HFB4). The synthesized compounds also exhibited promising pharmacokinetic and drug-likeness properties, as confirmed by an ADME prediction study [38]. 

A series of biphenylurea-indolinones along with their antiproliferative evaluation on two human cancer cell lines viz. MCF-7 and PC-3 using sulforhodamine B (SRB) colorimetric assay was disclosed by Eldehna et al. Among the synthesized compounds, **27** proved to be the most active hybrid, with an IC_50_ of 1.04 μM on MCF-7 cells being seven-fold more active than the reference drug, doxorubicin (IC_50_ = 7.30 ± 0.84 μM) (Figure 5) [39].

Gabr et al. synthesized a series of isatin-*β*-thiocarbohydrazones and evaluated their antiproliferative activities against cervical cancer (Hela) and kidney fibroblast cancer (COS-7) cell lines. Few hybrids exhibited excellent activities against Hela and COS-7 cell line, with **28** being most potent, exhibiting IC_50_s of 1.51and 2.19 μM against Hela and COS-7 cell lines, respectively (Figure 5). The promising compounds were further examined for in vivo antitumour efficacy against Ehrlich ascites carcinoma (EAC) in mice [40].

Althagafi et al. synthesized a series of *bis*-(indoline-2,3-dione) via reactions of isatin with 1,3-dibromopropane with subsequent condensation reaction with hydrazine derivatives. The antiproliferative activity of the synthesized hybrids was assayed on MCF-7 cells and revealed some compounds exhibiting IC_50_s ranging from 0.0028 to 0.028 μM, with compound **29** being the most potent, having an IC_50_ of 0.0028 μM on MCF-7 cancer cells (Figure 5) [41].

### 2.5. Isatin-Based Schiff’s Bases and Oximes

**Figure 6:**
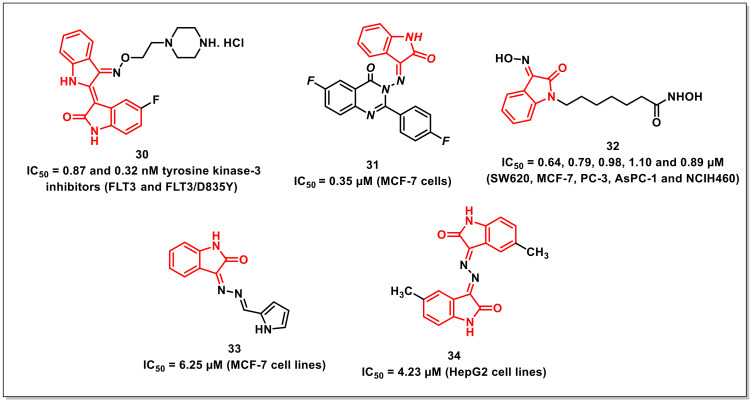
Antiproliferatives Schiff’s bases of isatins.

Jeong et al. described the synthesis of FMS-like receptor tyrosine kinase-3 (FLT3) inhibitors via the optimization of indirubin derivatives along with an evaluation of their biological and pharmacokinetic profiles. Among the synthesized hybrids, compound **30** displayed IC_50_s of 0.87 and 0.32 nM against FLT3 and FLT3/D835Y, respectively (Figure 6). Additionally, compound **30** inhibited MV4-11- and FLT3/D835Y-expressed MOLM14 cells, with GI_50_s of 1.0 and 1.87 nM, respectively. The compound exhibited oral bioavailability of 42.6% with substantial in vivo antitumour activity via oral administration of 20 mg/kg and a once-daily dosing schedule for 21 days in a mouse xenograft model [42].

Zayed et al. synthesized a series of isatin-fluoroquinazolinone hybrids and assayed for their in vitro antiproliferative activity on MCF-7 and MDA-MBA-231 cells. Among the synthesized hybrids, some exhibited broad spectrum anticancer activity. The hybrid **31** proved to be most potent among the series, with an IC_50_ of 0.35 μM on MCF-7 cells—better than the reference drug gefitinib (IC_50_ = 0.97 μM) (Figure 6). Additionally, EGFR and tubulin inhibition assays were carried out for the promising hybrids, and outstanding results were observed compared to the reference drugs. Molecular docking studies were further performed in order to delineate the mechanism of action of the promising compounds in the EGFR binding site [43]. 

Nam et al. designed and synthesized two series of isatin-3’-oxime and isatin-3’-methoxime-based hydroxamic acids as analogues of SAHA approved by the FDA for treating cutaneous T-cell lymphoma. Among the synthesized series, the compounds that either had or lacked a halogen (F or Cl) substituent at the 5th or 7th position on the isatin core exhibited hindrance against histone-H3 and histone-H4 deacetylation. In addition, compound **32** showed potent cytotoxicity against five cancer cell lines, with IC_50_s of 0.64, 0.79, 0.98, 1.10, and 0.89 μM against SW620, MCF-7, PC-3, AsPC-1, and NCIH460, respectively (Figure 6). A docking study performed with the selected compound **32** revealed that these compounds bind to HDAC8 with higher affinities than SAHA. Furthermore, the carbonyl oxygen of the isatin moiety in compound **32** was assessed to associate with the backbone amide through hydrogen bonds [44].

Dweedar et al. synthesized a series of indoline-2,3-dione hydrazones via treating indoline-2,3-diones with hydrazine to yield 3-hydrazonoindolin-2-ones, which were reacted with the appropriate aldehydes. From the biological assay results, it was found that some compounds showed notable activity, with IC_50_ values in the range of 6.25–25.8 μM, among which **33** gave the most extreme activity, with an IC_50_ of 6.25 μM (Figure 6). The docking analysis of the GSK-3b receptor for both indirubin-3’-oxime and our suggested compounds, taking **33** as representative example, showed that they interacted through hydrogen bonds [45]. 

Liang et al. synthesized symmetrical bis-Schiff bases of isatin via the condensation of natural or synthetic isatins with hydrazine and evaluated their in vitro and in vivo antitumour activities on five diverse human cancer cell lines (i.e., HeLa, SGC-7901, HepG2, U251, and A549). Compound **34** proved to be the most promising compound on HepG2 (IC_50_ = 4.23 μM) (Figure 6). Additionally, **34** considerably hindered the tumour development of HepS-bearing mice at a dose of 40 mg/kg. Mechanistically, **34** arrested the cell cycle at the G2/M stage, with downregulation of cyclin B1 and cdc 2 expression [46].

### 2.6. Metal Complexes of Isatin Containing Ligands

**Figure 7:**
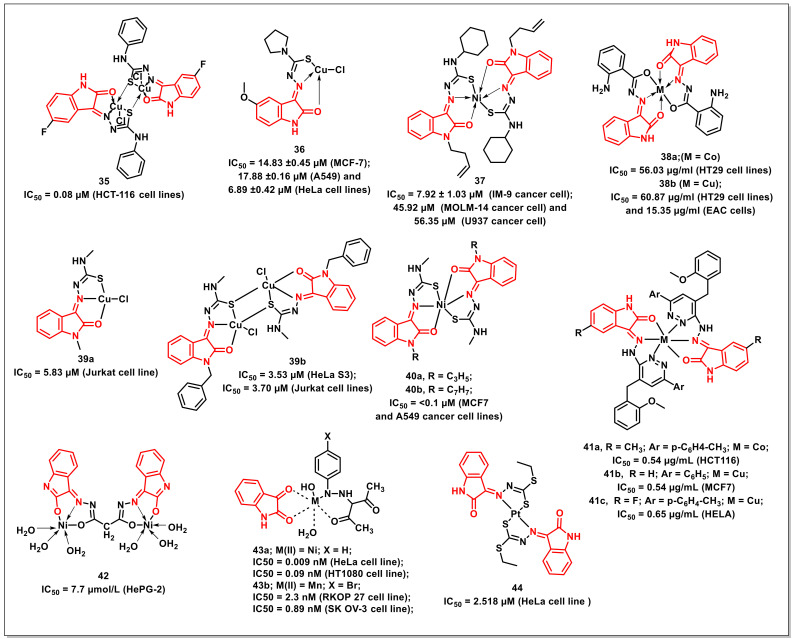
Metal complexes of isatins as promising antiproliferatives.

Ali et al. synthesized and characterized six tridentate Cu(II) complexes of thiosemicarbazone with isatin (CuL1-CuL6) and evaluated their antiproliferative activities. Gel electrophoresis indicated that these complexes could prompt the cleavage of plasmid DNA. It was revealed that the DNA cleavage by these complexes is concentration-dependent. CuL1 and CuL2 exhibited oxidative behaviour towards DNA, while CuL3–CuL6 complexes induced DNA cleavage via oxidative and hydrolytic pathways. In vitro antiproliferative activity on human colon cancer cell line (HCT-116) showed concentration-dependence with low IC_50_s (0.08–8.6 μM). The complex **35** proved to be most potent, with an IC_50_ of 0.08 μM against human colorectal cancer (HCT116) cells, which is more pronounced than the reference drug, 5-fluorouracil (IC_50_ = 7.3 μM) (Figure 7) [47].

Aneesrahman et al. synthesized 5-methoxy-isatin thiosemicarbazone ligands with different *N*-terminal substituents and their copper(II) complexes. The binding studies revealed their affinity to DNA/BSA with a high binding constant. The complex **36** displayed broad spectrum antiproliferative activities against MCF-7, A549, and HeLa, with IC_50_ values of 14.83 ± 0.45 μM, 17.88 ± 0.16 μM, and 6.89 ± 0.42 μM, respectively (Figure 7) [48].

Balachandran et al. synthesized *N*-substituted isatin thiosemicarbazone-based complexes along with an assessment of their antiproliferative profiles on a panel of four human cancer cells, HepG-2 (liver), MOLM-14 (acute monocytic leukemia), U937 (histiocytic lymphoma), and IM-9 (myeloma). Complex **37** displayed promising activity against IM-9 cells, with an IC_50_ of 7.92 ± 1.03 μM; however, it had no effect on HepG-2 cells (Figure 7). Its activity on MOLM-14 cells (IC_50_ = 45.92 μM) was significant, while good activity was observed on U937 cells (IC_50_ = 56.35 μM). Mechanistically, **37** accelerated apoptotic cell death in the IM-9 cells, causing cell cycle arrest at the G1 phase. It also downregulated Bcl-2 (b-cell lymphoma-2), upregulated Bax (bcl-2 associated X protein), released cytochrome c, and triggered caspases-3 in IM-9 cells [49].

Hunoor et al. reported the synthesis of Co(II), Ni(II), Cu(II), and Zn(II) complexes of isatin and 2-aminobenzoylhydrazide. The synthesized compounds were evaluated for their antiproliferative activities, targeting Ehrlich ascites carcinoma (EAC cells) and HT29 cell line. Cu(II) complex **38b** exhibited a 50% inhibition of EAC cells at 15.35 μg/mL while its activity on HT29 cells was 60.87 μg/mL (Figure 7) [50].

Haribabu et al. synthesized and assayed mono- and bi-nuclear copper(II) complexes containing *N*-substituted isatin-thiosemicarbazones for their antiproliferative activity. Complex **39** showed promising cytotoxic activity against the Jurkat (leukaemia) and HeLa S3 (cervical) cell lines, with IC_50_s of 5.83 and 3.53 μM, respectively. The complex **39** was nine-fold more active than cisplatin against HeLa S3 and Jurkat cell lines, while it proved to be non-cytotoxic to the IMR90 cell line, as indicated by its high IC_50_ values (>100 μM) (Figure 7) [51].

The group further extended the approach and synthesized a series of nickel(II) complexes [Ni(L)_2_] of *N*-substituted isatin thiosemicarbazones. In vitro cytotoxicity evaluation revealed substantial activities on human breast (MCF7) and lung (A549) cancer cells, with the best outcomes being recorded for complexes **40a** and **40b**, exhibiting IC_50_ < 0.1 μM (Figure 7). Complexes **40a** and **40b** showed a stronger DNA binding affinity than the other complexes, which could be attributed to the presence of benzyl and allyl groups [52].

Kandileet al. synthesized the hydrazone ligands (HL) from 5-substituted isatins and 1-(4-(2-methoxybenzyl)-6-arylpyridazin-3-yl) hydrazines and their corresponding complexes with Co(II) and Cu(II), along with an assessment of their antiproliferative activities. The cobalt complex (**41a**) had the highest activity on the colon cancer (IC_50_ = 0.54 μg/mL), while the copper complex (**41b**) proved to be the most effective on the breast cancer cell line (IC_50_ = 0.54 μg/mL) (Figure 7). The copper complex (**41c**) exhibited an IC_50_ of 0.65 μg/mL against cervical tumour cell lines [53].

Youssefet al. synthesized and characterized the ligand (1*Z*,3*Z*)-*N*’1,*N*’3-bis((*E*)-2-hydroxy-3*H*-indol-3-ylidene)malonohydrazonic acid and its Ni(II) complexes. These complexes were tested for their in vitro cytotoxicity against hepatocellular carcinoma human tumour cells (HePG-2). It was found that the Ni(II) complex (**42**) had very strong activity against HePG-2, with an IC_50_ of 7.7 μM (Figure 7) [54]. 

Osmanet al. synthesized Ni(II), Co(II), and Mn(II) complexes of 3-(2-arylhydrazono)acetylacetone and isatin. The complex **43a** (IC_50_ = 0.009 nM) was more potent on the HeLa (cervical) cell line than the conventional medication, tamoxifen (IC_50_ = 0.114 nM) (Figure 7). Complexes **43a** and **43b** proved to be active against HT1080 (fibrosarcoma) (IC_50_ = 0.09 nM) and ovarian carcinoma (SK OV-3) (IC_50_ = 0.89 nM) cells, respectively. Complex **43b** (IC_50_ = 2.30 nM) proved to be more potent than the standard drug, capecitabine (IC_50_ = 4.33 nM), in the inhibiting the proliferation of the colon cancer (RKOP 27) cell line [55].

Yekke-ghasemiet al. synthesized platinum complexes of diethyl methylene (1*Z*,1’*Z*)-bis(((*E*)-2-oxoindolin-3-ylidene)carbonohydrazonodithioate). Two human cancer cell lines, HeLa and MCF-7, were used in order to investigate the cytotoxicity of synthesized complexes. The antiproliferative activities suggested that the coordination of ligand to metal ions has a significant influence on cytotoxicity, with complex **44** being more active than cisplatin against the HeLa cell line (IC_50_ = 2.518 μM) (Figure 7) [56].

### 2.7. Miscellaneous Compounds Containing Isatin Core

**Figure 8:**
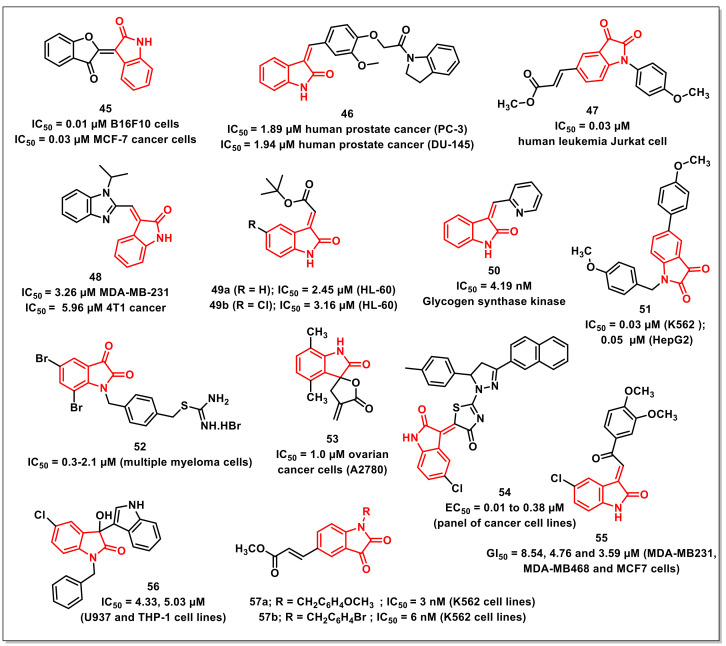
Miscellaneous examples of isatin-based compounds with promising antiproliferative/anticancer activities.

Evdokimovet al. synthesized a series of indirubin analogues and isatin-based heterocycles via condensation with active methylenes. Most of the synthesized compounds displayed antiproliferative activities in low micro molar concentrations. The most promising hybrid among the series, **45**, exhibited good activities against apoptosis resistant glioblastoma (GBM) and non-small cell lung cancer (NSCLC) as well as apoptosis-sensitive murine melanoma (B16F10) cells (Figure 8). The hybrid **45** was more active on the apoptosis-sensitive cancer cell lines, exhibiting nanomolar activity against B16F10 cells (IC_50_ ˂ 0.01 μM) and MCF-7 cancer cells (IC_50_ = 0.3 μM) [57].

A series of (*Z*)-3-(3′-methoxy-4′-(2-amino-2-oxoethoxy)benzylidene)indolin-2-ones were synthesized and evaluated for their antiproliferative activities on a panel of prostate, breast, and noncancer cells by Senwaret al. Compound **46** proved to be the most promising of the synthesized scaffolds, being selective on prostate cancer cells with IC_50_s of 1.89 and 1.94 μM on PC-3 and DU-145 cells (Figure 8). The treatment of PC-3 cells with **46** resulted in the inhibition of in vitro cell migration through the disruption of F-actin proteins, while flow cytometric studies have revealed cell cycle arrest at the G2/M phase in a dose-dependent manner. Further, the collapse of mitochondrial membrane potential and increase in intracellular Ca^2+^ levels were responsible for the apoptotic effect of **46** on PC-3 cell lines [58].

Tenget al. disclosed the synthesis and antiproliferative evaluation of a series of di- or trisubstituted isatins on the human tumour cell line Jurkat using MTT assay. The presence of benzyl-substituent at N-1, *trans*-2-(methoxycarbonyl)ethen-1-yl at C-5, and an intact carbonyl functionality at the C-3 position of the isatin ring greatly enhanced the cytotoxic activity. Among the synthesized scaffolds, compound **47** proved to be most cytotoxic, with an IC_50_ of 0.03 μM, >330-fold higher than the parent molecule (Figure 8). Compound **47** exhibited antiproliferative activities via inducing mitochondrial apoptosis, as confirmed by cell morphology changes, a annexin-V/PI-staining study, and the activation of caspase-3 [59]. 

Sharmaet al. reported the synthesis and antiproliferative activities of a series of (((*E*)-benzo[*d*]imidazol-2-yl)methylene)indolin-2-ones on a panel of human cancer cell lines along with normal breast epithelial cells. The preliminary evaluation studies revealed that some of the synthesized hybrids were active on the tested cancer cell lines and safer when tested on normal human cell lines. The most promising compound among the series, **48**, displayed good cytotoxic activity on MDA-MB-231 and 4T1 cancer cells, with IC_50_s of 3.26 and 5.96 μM, respectively (Figure 8). Treating MDA-MB-231 cells with **48** resulted in in vitro cell migration inhibition via the disruption of the F-actin protein assembly along with the arresting of cells in the G0/G1 phase. Further, the collapse of mitochondrial membrane potential and the elevated generation of intracellular ROS was also observed on treatment with **48**. Compound **48** also led to the activation of caspase-3 and an increased expression of cleaved PARP, confirmed by Western blotting analysis [60]. 

Songet al. synthesized a series of (2*E*)-(2-oxo-1,2-dihydro-3*H*-indol-3-ylidene)acetates and assayed it on a panel of cancer cell lines. The results indicated that two compounds viz. **49a** and **49b** were promising, with IC_50_s of 2.45 μM and 3.16 μM against HL-60, respectively (Figure 8). Mechanistically, compound **49a** increased reactive oxygen species level via inhibition of TrxR and induced apoptosis via activation of bax and cleaved-caspase 3 in HCT116 cells, as confirmed by Western blotting studies. SAR studies confirmed the presence of double bond as important for the cytostatic activities of the synthesized Michael acceptors [61]. 

Lozinskaya and co-workers have reported the synthesis of 3-arylidene-2-oxindoles as Glycogen synthase kinase 3β (GSK-3β) inhibitors. The most promising compound among the synthesized series, **50,** was found to inhibit GSK-3β, with an IC_50_ of 4.19 nM (Figure 8). Compound **50** was found to effectively occupy the hydrophobic cleft of the allosteric binding site. The pyridine moiety is oriented toward the solvent-accessible region of the pocket, exposing it to polar substituents that influence the compound’s solubility and pharmacokinetic properties [62].

Zhanget al. synthesized a series of *N*-1 benzyl substituted 5-arylisatins and evaluated their antiproliferative activities on human leukemia K562 and liver cancer HepG2 cells using an MTT assay. The presence of *N*-benzyl and C-5 phenyl substituents along with an intact carbonyl functionality at the C-3 position proved to be important for good antiproliferative activities. Compound **51** proved to be the most potent among the series, exhibiting the highest antiproliferative activity against K562 cells, with an IC_50_ of 0.03 μM, while an IC_50_ of 0.05 μM was observed on HepG2 cells (Figure 8). The morphological results and flow cytometry analysis revealed that the cell cycle arrest resulted in a time- and dose-dependent manner. Additionally, the results of cell tube formation have shown the inhibition of the angiogenesis of HUVEC cells by **51** via a dose-dependent inhibitory effect [63].

Dinavahiet al. synthesized a series of isatin-based ALDH-isoform inhibitors. The synthesized compounds exhibited ALDH inhibitory IC_50_s ranging from 230 to >10,000 nM (ALDH1A1); 939 to >10,000 nM (ALDH2); and 193 nM to >10,000 nM (ALDH3A1). Most compounds among the series exhibited IC_50_s ranging from 2.1 to 5.7 μM on melanoma cells, 2.5 to 5.8 μM for colon cancer cells, and 0.3 to 4.7 μM for multiple myeloma cells. The most potent compound, **52**, exhibited IC_50_s ranging from 0.3 to 2.1 μM for multiple myeloma cells (Figure 8). Additionally, the compounds resulted in enhanced ROS activity, lipid peroxidation, and toxic aldehyde accumulation, resulting in increased apoptosis and cell cycle arrest in the G2/M phase [64].

Ranaet al. designed, synthesized, and evaluated a series of α-methylene-γ-butyrolactones for their antiproliferative activities. Among the synthesized hybrids, a spirocyclic analogue proved to be an inhibitor of TNFα-induced NF-κB activity, cancer cells, and tumour growth in an ovarian cancer model. Another repetition of the biological assay identified analogue **53**, which exhibited an IC_50_ of 1.0 μM, being 13-fold more active than parthenolide and 20-fold more active than ibrutinib against ovarian cancer cells, namely A2780 cells (Figure 8). Compound **53** exhibited IC_50_s of 3.2, 0.9, and 1.2 μM against HeLaGFP, MiaPaCa2, and SW480 cancer cells [65].

Meledduet al. synthesized a series of isatin-dihydropyrazole hybrids so as to assess their antiproliferative activities on a panel of cancer cell lines. Among the synthesized compounds, four hybrids displayed interesting activity and were further assayed to determine their EC_50_ values. Compound **54** proved to be the most promising among the synthesized series and exhibited EC_50_ values ranging from 0.01 to 0.38 μM (Figure 8) [66]. 

Karthikeyanet al. designed and synthesized 3-(2-oxo-2-phenylethylidene)indolin-2-ones, incorporating isatin and chalcone as the pharmacophores. The compounds were assessed for anticancer activity against three breast cancer cell lines. Most of the compounds showed promising anticancer activity (<20 μM) against the contemplated cell lines. Despite the structural resemblances that isatin-linked chalcones share with known kinase inhibitors, the mechanism of antibreast cancer activity shown by synthesises compounds might be kinase-independent. Compound **55**, bearing a chloro substituent at C-5 of the isatin ring and 3,4-dimethoxy substituents on the phenyl ring of chalcone, was the most potent, with GI_50_ values of 8.54, 4.76, and 3.59 μM against MDA-MB231, MDA-MB468, and MCF7 cells, respectively (Figure 8) [67]. 

Prathimaet al. synthesized a series of 3-indolyl-3-hydroxy oxindole derivatives to evaluate their cytotoxic potential on a panel of cancer cell lines. Biological results revealed that the synthesized scaffolds displayed noteworthy antiproliferative activities against leukemia (U937, THP-1), lung (A549), and breast cancer cell lines (MCF7). The order of susceptibility of human cancer cells towards the synthesized oxindoles was MCF7 > U937 > A549 > THP1. Compound **56** has shown excellent cytotoxic activity, IC_50_ = 4.33 and 5.03 μM, against U937 and THP-1 cell lines, respectively (Figure 8) [68].

Hanet al. reported the synthesis of di- or tri-substituted isatins along with their in vitro antiproliferative evaluation on three human tumour cell lines, K562, HepG2, and HT-29, using an MTT test. SAR studies revealed that the presence of 1-benzyl and 5-[*trans*-2-(methoxycarbonyl)ethen-1-yl], along with an intact carbonyl at the C-3 positions, were prerequisites for good cytotoxic activity. In acrylate-containing isatin analogues, the size and orientation for N-substitution is important for their anticancer activity. The compounds **57a** and **57b** were identified as the two promising compounds among the synthesized series on human leukemia K562 cells, with an IC_50_ of 3 and 6 nM, respectively (Figure 8) [69]. 

## 3. Antimycobacterial/Tubercular Activities of Isatin-Based Scaffolds

After the outbreak of COVID-19, TB is expected to be the second leading cause of death from a single infectious agent. In 2020, the WHO African and South-East Asia regions accounted for over 84% of HIV-negative TB deaths and 85% of all TB deaths among HIV-negative and HIV-positive adults. [70]. India accounted for 38% of global TB deaths among HIV-negative people and 34% of the total number of TB deaths among HIV-negative and HIV-positive people combined [71]. The rapid emergence of multi drug resistant (MDR) tuberculosis, combined with *Mycobacterium tuberculosis* (*Mtb*)’s prominent ability to enter a dormant state, known as latent TB infection, poses significant challenges for tuberculosis control [72,73]. As a result, the identification of novel targets and the development of new agents capable of combating drug-resistant tuberculosis and latent tuberculosis infection remain urgent priorities [74].

### 3.1. Ciprofloxacin-Isatin and Moxifloxacin-Isatin Hybrids

**Figure 9:**
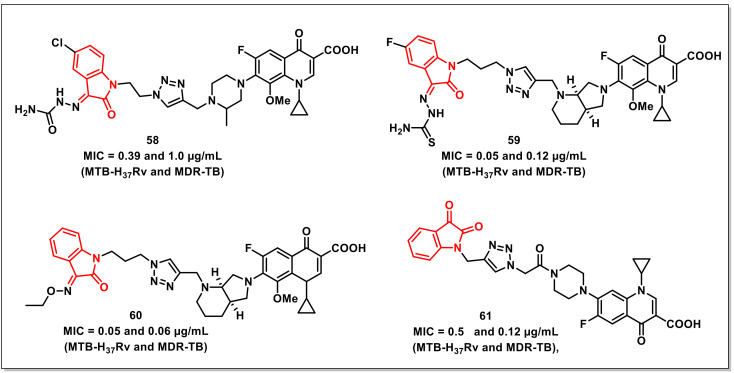
Ciprofloxacin and moxifloxacin-based isatins as antimycobacterials.

Xuet al. designed, synthesized, and evaluated a series of 1*H*-1,2,3-triazole-tethered ciprofloxacin-isatin (thio) semicarbazide/oxime hybrids for their in vitro antimycobacterial activities against *M. tuberculosis* (*Mtb*) H_37_Rv and MDR-TB, as well as their cytotoxicity. Most of the synthesized scaffolds displayed good to excellent activities against *Mtb* H_37_Rv and MDR-TB (MIC ranging from 0.39 to 16 μg/mL), with activities being reportedly better than in the parent 8-OMe CPFX. The most active of the synthesized hybrids, **58**, with an MIC of 0.39 and 1.0 μg/mL, respectively, was two to eight-fold more potent than CPFX (MIC: 3.12 and 4.0 μg/mL, respectively) and 8-OMe CPFX (MIC: 1.56 and 2.0 μg/mL) against *Mtb* H_37_Rv and MDR-TB (Figure 9). SAR studies have shown that the inclusion of chloro at the C-5 position and thiosemicarbazone/semicarbazone at the C-3 position of isatin improved the activity on both *Mtb* H_37_Rv and MDR-TB strains. Further, the synthesized hybrids exhibited acceptable cytotoxicity (CC_50_: 16–64 μg/mL), though they were more toxic than the parent 8-OMe CPFX (CC_50_: 64 μg/mL) in the VERO cell line [75]. 

The authors have further extended the above study by synthesizing a series of 1*H*-1,2,3-triazole-tethered isatin-moxifloxacin hybrids, evaluating their antimycobacterial activities on *Mtb* H_37_Rv and MDR-TB and cytotoxicity on VERO cells. The synthesized hybrids (MIC: 0.05–2.0 μg/mL) displayed excellent activities on the H_37_Rv strain and on MDR-TB. The hybrid **59**, with an MIC of 0.05 and 0.12 μg/mL, proved to have compatible activities to that of MXFX (MIC: 0.10 and 0.12 μg/mL), RIF (MIC: 0.39 and 32 μg/mL), and INH (MIC: 0.05 and >128 μg/mL) on the tested strains (Figure 9). SAR studies implied that the inclusion of -Cl and -F substituents at the C-5 position of the isatin ring improved the antimycobacterial activity, while the hybrids having a semicarbazone moiety at the C-5 position proved to be more active than the corresponding thiosemicarbazone analogs. The synthesized hybrids (CC_50_: 2–8 μg/mL) were, however, more cytotoxic than the parent MXFX (CC_50_: 128 μg/mL) drug [76]. 

Yanet al. synthesized a series of 1*H*-1,2,3-triazole-tethered isatin-moxifloxacin hybrids and assayed them for their in vitro antimycobacterial activities against drug sensitive and multidrug-resistant *Mtb*, while the cytotoxicity was assessed on VERO cells. Most of the synthesized compounds exhibited excellent activities, with MICs in the range from 0.05 to 2.0 μg/mL. The most potent hybrid, **60** (MIC of 0.05 and 0.06 μg/mL on *Mtb* H_37_Rv and MDR-TB), was two to eight-fold more potent than the reference drugs, moxifloxacin, and rifampicin on H_37_Rv, while 2 to >2048-fold more active than moxifloxacin, rifampicin, and isoniazid on MDR-TB (Figure 9). The synthesized hybrids were, however, more cytotoxic than the moxifloxacin against the VERO cell line [77]. 

A series of amide-tethered ciprofloxacin-isatin hybrids were synthesized by Chen and co-workers, who evaluated their in vitro antimycobacterial activity. The synthesized hybrids displayed excellent in vitro activity on both H_37_Rv and MDR strains, with MIC values ranging from 0.12 to 32 μg/mL. Hybrid **61** proved to be most active among the synthesized series, with an MIC of 0.5 μg/mL (H_37_Rv) and 0.12 μg/mL (MDR-TB), comparable to that of isoniazid (0.12 μg/mL) and rifampicin (0.25 μg/mL) (Figure 9) [78].

### 3.2. Spiro-Isatin Derivatives

**Figure 10:**
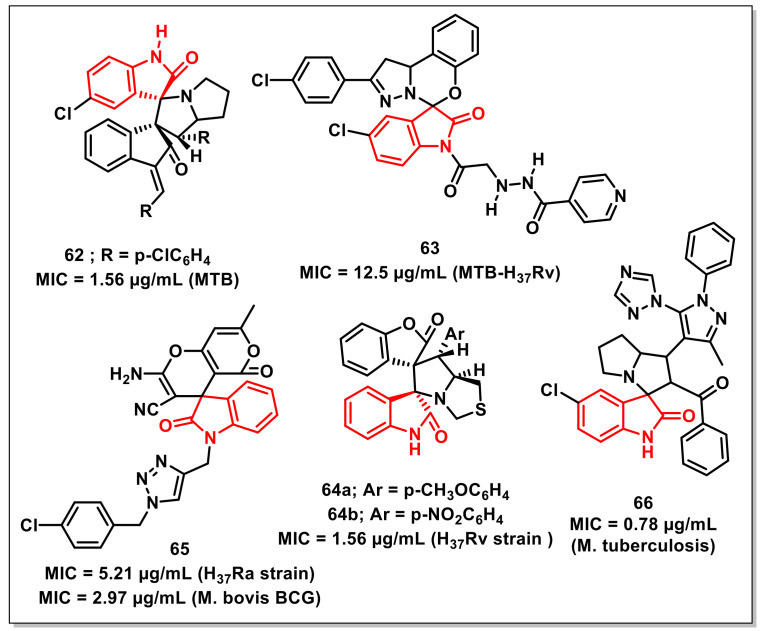
Spiro-isatins as promising antimycobacterials.

Rouatbiet al. reported the synthesis and antimycobacterial evaluation of spirooxindoles via 1,3-dipolar cycloaddition of azomethine ylides, generated in situ from isatin or acenaphthenequinone with L-proline and (*E,E*)-1,3-bis(arylidene)indan-2-ones. The synthesized compounds exhibited MIC values in the range of 1.56–25 μg/mL. Some of the synthesized compounds exhibited better activities on *Mtb* than the antiTB drug pyrazinamide (MIC 6.25 μg/mL). The synthesized compounds, however, were less potent than rifampicin and isoniazid. Compound **62** exhibited maximum activity, with an MIC of 1.56 μg/mL (Figure 10) [79].

Boradet al. designed and synthesized a library of isoniazid-spirooxindole hybrids to evaluate their in vitro antimycobacterial activity on *Mtb* H_37_Rv and MDR-TB. Compound **63** proved to be most active among the series, with an MIC value 12.5 μg/mL (Figure 10). Molecular docking and molecular dynamic studies have revealed the binding modes of the synthesized compounds in the active site of isoniazid-resistant enoyl-ACP(COA) reductase. Compound **63,** with the highest MIC value, showed two crucial H-bonding with Gly96 (1.83 and 1.91 Å) through the -NH and -CO of the side chain [80]. 

Mhiriet al. synthesized a series of dispirooxindolopyrrolidines and dispirooxindolopyrrolothiazoles via a three-component 1,3-dipolar cycloaddition of (Z)-3-arylidenebenzofuran-2-ones, substituted isatins, and α-aminoacids. X-ray diffraction analysis was used to confirm the stereochemistry of the synthesized spiro adducts. The synthesized scaffolds were assessed in vitro on the *Mtb* H_37_Rv strain along with cytotoxicity assessment on RAW 264.7 cells. Among these, the potent compounds displayed antiTB activities, with MIC values ranging from 1.56 to 6.25 μg/mL. Specifically, dispirooxindolopyrrolothiazoles, **64a** and **64b**, were proved to be the most promising compounds, with an MIC of 1.56 μg/mL (Figure 10) [81]. 

Chavanet al. synthesized a series of 1*H*-1,2,3-triazole-linked spirochromenes via a single-pot, five-component reaction between *N*-propargyl-isatins, malononitrile, dimedone, aryl/alkyl halides, and sodium azide, utilizing cellulose-upheld CuI nanoparticles (Cell-CuI NPs) as a heterogeneous catalyst. The synthesized scaffold, **65**, exhibited promising antimycobacterial activity on *Mtb* H_37_Ra and *M. bovis* BCG, with MIC values of 5.21 and 2.97 μg/mL, respectively (Figure 10). The SAR studies revealed the dependence of activity on the nature of the substituents on the phenyl rings, with a preference for halogen [82]. 

Pogakuet al. designed and synthesized 1,2,4-triazol-1-yl-pyrazole-based spirooxindolopyrrolizidines, along with their antimycobacterial evaluation. Among the synthesized scaffolds, few compounds displayed comparable activity to ethambutol (MIC: 1.56 μg/mL), while the most potent compound of the series, **66**, exhibited an MIC of 0.78 μg/mL—a better value than the standard drug ethambutol (Figure 10) [83].

### 3.3. Schiff’s Bases and Oximes of Functionalized Isatins

**Figure 11:**
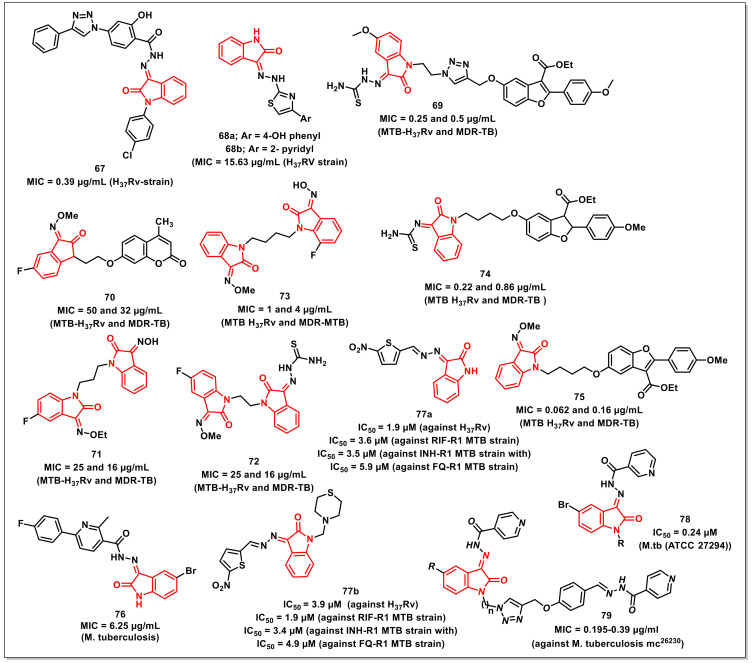
Schiff bases and oximes of isatin with antimycobacterial potential.

Abdu-Allahet al. synthesized a series of 1*H*-1,2,3-triazolylsalicylhydrazones of isatins using para-amino salicylic acid (PAS) as the core scaffold. The synthesized scaffolds were assayed for their antimycobacterial activities on *Mtb* H_37_Rv and exhibited good to high activity, with an MIC of 0.39-1.5 μg/mL. The promising compound among the series, **67,** proved to be ten-fold more potent than PAS and equipotent to rifampicin (MIC = 0.39 μg/mL), with a selectivity index of >128 (Figure 11). Additionally, the synthesized compound also proved to be active against persistent forms of mycobacteria comparable to standard drugs in the nutrient starvation model. A study was also conducted in order to explain the observed activity and to serve as a tool for further development [84].

Yurttaşet al. synthesized a series of isatin-thiazole-based hydrazones and screened their antimycobacterial activity against the H_37_Rv strain. The synthesized compounds **68a** and **68b**, having 4-hydroxyphenyl and 2-pyridyl moiety, respectively, exhibited significant activity (MIC = 15.63 μg/mL) two-fold greater than isoniazid (MIC = 31.25 μg/mL) (Figure 11) [85].

Gaoet al. reported the synthesis and in vitro antimycobacterial evaluation of benzofuran-isatin hybrids on both drug-susceptible and MDR *Mtb* strains, while the cytotoxicity was determined on VERO cells. The results of antimycobacterial evaluation studies revealed that the synthesized hybrids have shown substantial activities against H_37_Rv and MDR-TB, with MIC values ranging from 0.25 to 8 μg/mL. The non-cytotoxic hybrid **69** proved to be the most active, with MIC values of 0.25 and 0.5 μg/mL on H_37_Rv and MDR-TB, respectively, being two-fold more active than rifampicin and isoniazid on MDR-TB (Figure 11). The promising compound **69** was further assessed for its metabolic stability and in vivo pharmacokinetic profiles [86]. 

The authors further extended the work towards the synthesis of a series of isatin-coumarin hybrids for assessing their in vitro antimycobacterial activities on *Mtb* H_37_Rv and MDR-TB. The synthesized hybrids displayed MICs ranging from 32 to 256 μg/mL against the tested strains. The most promising of the synthesized hybrids, **70**, exhibited MIC values of 50 and 32 μg/mL on H_37_Rv and MDR-TB, respectively, being two and four-fold more potent than rifampicin and isoniazid on MDR-TB (Figure 11) [87].

A series of propylene-tethered bis-isatin derivatives were synthesized and evaluated for their in vitro antimycobacterial activities by Huaet al. The synthesized hybrids displayed considerable antimycobacterial activities on H_37_Rv and MDR-TB, with MIC values ranging from 16–256 μg/mL. In particular, compound **71**, with MIC values of 25 and 16 μg/mL on H_37_Rv and MDR-TB, respectively, proved to be the most promising hybrid (Figure 11) [88]. 

Xuet al. reported the synthesis of a series of homo and hetero-nuclear bis-isatins for evaluating their in vitro antimycobacterial activities against *Mtb* H_37_Rv and MDR-TB. The synthesized hybrids displayed moderate to good activities against the tested strains, with MIC values ranging from 16 to 256 μg/mL. In particular, the bis-isatin **72** proved to be the most active against H_37_Rv and MDR-TB, with MIC values of 25 and 16 μg/mL, respectively (Figure 11) [89]. 

Liuet al. synthesized and evaluated a series of butylene-tethered heteronuclear 7-fluoroisatin-isatin scaffolds for their antimycobacterial activity, cytotoxicity, and inhibitory activity on *Mtb* DNA gyrase. Most of the synthesized compounds were active on the H_37_Rv and MDR-TB strains, with some being more potent than the standard drugs, such as isoniazid, rifampicin, and ethambutol. Compound **73** proved to be the most active compound of the series, with MIC values of 1 and 4 μg/mL on H_37_Rv and MDR-TB, respectively (Figure 11). Additionally, **73** also displayed good toxicological profiles and promising inhibitory activity against *Mtb* DNA gyrase [90]. 

Zhang synthesized a library of benzofuran-isatin-hydroxylimine/thiosemicarbazide hybrids and evaluated them for their in vitro antimycobacterial activities. The synthesized hybrids exhibited considerable activities on the tested *Mtb* strains, with acceptable cytotoxicity. The hybrid **74** proved to be the most active one, being >4.8 and >51-fold more active than the first line antiTB drugs, RIF and INH, on the drug-sensitive H_37_Rv and MDR-TB isolates, with MIC values of 0.22 and 0.86 μg/mL, respectively (Figure 11) [91]. 

Gaoet al. reported the synthesis of the antimycobacterial and antibacterial evaluation of benzofuran-isatin-imine hybrids tethered via different alkyl chain lengths. The synthesized hybrids displayed considerable in vitro antiTB activity, with MIC values ranging from <0.016 to 0.218 μg/mL and 0.062 to 14.15 μg/mL on drug-sensitive strains. The antibacterial activities ranged from 0.25 to 64 μg/mL (Gram-positive) and 0.06 to 16 μg/mL (Gram-negative) strains. The most active hybrid among the synthesized compounds, **75** (MIC = 0.062 and 0.16 μg/mL for MTB H_37_Rv and MDR-TB, respectively), was >4.8 and ≥48-fold more active than the first line antiTB drugs, RIF and INH, on H_37_Rv and MDR-TB isolates, respectively (Figure 11). Further, **75** also displayed promising antibacterial activities, with MIC values of ≤1 μg/mL against most of the tested Gram-negative and Gram-positive pathogens [92].

Eldehnaet al. synthesized a series of nicotinic acid hydrazides and assayed for their antimycobacterial activity. The activity results revealed that the synthesized isatin-hydrazides were more active than the parent hydrazides. SAR studies revealed the lipophilicity as a crucial element for accounting for antimycobacterial activity, with a preference of halogen over the hydrogen as substituent. Compound **76**, with a bromo substituent at the C-5 position of the isatin core, displayed an MIC of 6.25 μg/mL (Figure 11) [93].

Karunanidhiet al. synthesized and characterized novel isatin hydrazones and their thiomorpholine-tethered analogues. Under level-I testing, all of the synthesized compounds were assayed for antimycobacterial activity against the H_37_Rv strain of *Mtb*. The most active compounds, **77a** and **77b**, exhibited IC_50_s of 1.9 and 3.9 μM, respectively (Figure 11). These compounds were then investigated against five drug-resistant strains of *Mtb*, comprising isoniazid-resistant strains (INH-R1 and INH-R2), rifampicin-resistant strains (RIF-R1 and RIF-R2), and a fluoroquinolone-resistant strain (FQ-R1). Interestingly, **77a** and **77b** were shown to be the most efficacious compounds against the RIF-R1 MTB strain, with IC_50_ values of 3.6 μM and 1.9 μM, respectively, followed by the INH-R1 MTB strain, with IC_50_ values of 3.5 μM and 3.4 μM, respectively. With an IC_50_ of 5.9 μM and 4.9 μM, respectively, the lead compounds **77a** and **77b** demonstrated effective suppression against the FQ-R1 MTB strain, indicating broad-spectrum efficacy. The electron-withdrawing (NO_2_) substituent on the electron-rich 5-membered heterocyclic system contributed significantly towards the achievement of enhanced antimycobacterial potency in both **77a** and **77b** [94].

Elsayedet al. reported the design and synthesis of isatin-nicotinohydrazide hybrids as promising antimycobacterial and antibacterial agents. A drug-susceptible *Mtb* strain (ATCC 27294) was used to evaluate the target hybrids’ antitubercular activity, and hybrid **78** was shown to be as effective as INH, with an MIC of 0.24 μM (Figure 11). In terms of the structure–activity relationship, it was discovered that the *N*-substitution and the presence of a halogen at the C-5 position of the oxindole ring impacted the antimycobacterial activity of halogen-bearing compounds. Compounds with a Br substituent have higher activity than Cl-substituted and -unsubstituted compounds [95].

Johansenet al. designed and synthesized, isatin-mono/bis-isoniazid hybrids with potential antimycobacterial activity. Compound **79** displayed high activity against drug-susceptible *Mtb*, with MICs ranging from 0.195 to 0.39 μg/mL (Figure 11). Moreover, these compounds were found to be well tolerated on Vero kidney cells at high concentrations (200 μg/mL), resulting in high selectivity indices. These compounds significantly suppressed mycolic acid biosynthesis and inhibited FAS-II components without affecting FAS-I. Mechanistically, these compounds shared a similar mode of action as INH, i.e., they required KatG in order to exhibit biological effects. However, the synthesized compounds were surprisingly more bactericidal and displayed a delayed resistance development compared to the standard drug INH when tested on the bacterial loads [96].

### 3.4. Ferrocene-Tethered Isatin Hybrids

**Figure 12:**
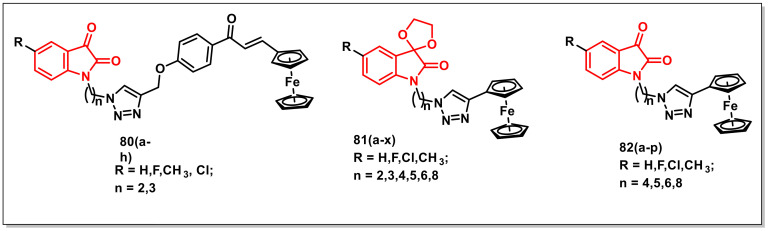
Ferrocene-tethered isatins as antimycobacterials.

Kumaret al. disclosed the synthesis and antimycobacterial evaluation of isatin-ferrocene and isatin-ferrocenyl chalcone hybrids obtained via Cu-promoted click reaction. Antimycobacterial SAR revealed the reliance of activity on the C-5 substituent of the isatin ring and the length of the alkyl chain, with a preference for halogen substituents (F, Cl) and a propyl chain length. The inclusion of a chalcone core among the isatin-ferrocene hybrids, **80a–h,** enhanced the antimycobacterial activities irrespective of the nature of the substituent at the C-5 situation (Figure 12) [97]. 

The authors further extended the work and synthesized a series of 1*H*-1,2,3-triazole-linked spiroisatin-ferrocene and isatin-ferrocenes. The synthesized hybrids **81a–x** and **82a–p** were examined for their antimycobacterial properties, showing improvement in activity profiles with the inclusion of a ferrocene core in contrast to their organic precursors (Figure 12). The observed SAR confirmed the independence of the length of the alkyl chain, nature of the substituent at C-5, and the presence of either a keto-carbonyl or ketal unit at the C-3 position of the isatin ring on antimycobacterial activities [98].

### 3.5. Miscellaneous Isatin Derivatives with Antimycobacterial Potential

**Figure 13:**
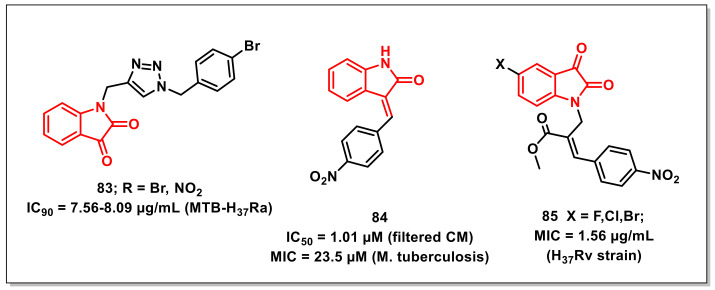
Miscellaneous examples of isatin-based scaffolds as antimycobacterials.

Shaikhet al. synthesized a series of triazole-based isatins using click reaction and evaluated their antimycobacterial activities against *Mtb* H_37_Rv. Compound **83** exhibited maximum antimycobacterial activity, with IC_90s_ of 7.56–8.09 μg/mL against H_37_Rv (Figure 13). Cytotoxicity studies proved that the promising scaffolds have low cytotoxicity on human cancer cell lines A549 and PANC-1. Molecular docking studies have shown the high affinity of the synthesized compounds towards cytochrome P450 lanosterol 14α-demethylase via van der Waals interactions with amino acid residues. Additionally, ADME parameters of the synthesized compounds confirmed their good drug-like properties [99].

Jean kumaret al. demonstrated the utilization of structure-based e-pharmacophore modelling to recognize the small molecule inhibitors of the *Mtb* chorismite mutase (CM) enzyme. Some isatin subsidiaries were synthesized and assessed in vitro for their ability to restrain MTB CM and activity against *M. tuberculosis*. Compound 3-(4-nitrobenzylidene)indolin-2-one, or **84**, proved to be the most promising and noncytotoxic among the synthesized hybrids, exhibiting an IC_50_ of 1.01 μM for filtered CM and an MIC of 23.5 μM on *M. tuberculosis* (Figure 13) [100].

Kumaret al. synthesized a series of Baylis–Hillman adduct-derived, *N*-cinnamyl-substituted isatin derivatives and assessed for their antitubercular and anticancer activities. Most of the synthesized compounds were more potent than Pyrazinamide (50.77 μg/mL) and less potent than Rifampicin (0.36 μg/mL) when tested on the *M. tuberculosis* H_37_Rv strain ATCC27294. Substitution of the halogen on the phenyl ring of the cinnamyl moiety with nitro **(85)** improved the antimycobacterial activities (1.56 μg/mL), which is comparable to that of INH (Figure 13) [101].

## 4. Antiplasmodial/Malarial Activities of Isatin-Based Scaffolds

*P. falciparum*, *P. vivax*, *P. ovale*, *P. malariae*, and *P. knowlesi* are members of the *Plasmodium* family of protozoan parasites that cause malaria. *P. falciparum* and *P. vivax* are the most virulent and are primarily responsible for the disease’s morbidity and mortality [102]. An estimated 241 million malaria cases were reported in 85 malaria-endemic countries in 2020, an increase from 227 million in 2019, with the WHO African Region accounting for the majority of the increase. The WHO African Region accounted for nearly 95 percent of all cases in 2020, with an estimated 228 million cases. India accounted for 83 percent of the cases in the Southeast Asian region. Malaria deaths increased by 11% in 2020 compared to 2019 to an estimated 627,000; of the additional 69,000 deaths, an estimated 47,000 (68%) were caused by service disruptions during the COVID-19 pandemic [103]. The continuous evolving drug resistance to both the conventional (quinolines) as well as contemporary (Artemisinin combination therapy) drugs has exacerbated the need for identifying new entities with promising antiplasmodial activities. A number of reports have shown the potential of isatin-based compounds as promising antiplasmodials [104,105]. 

### 4.1. Isatin-7-Chloroquinoline Hybrids

**Figure 14:**
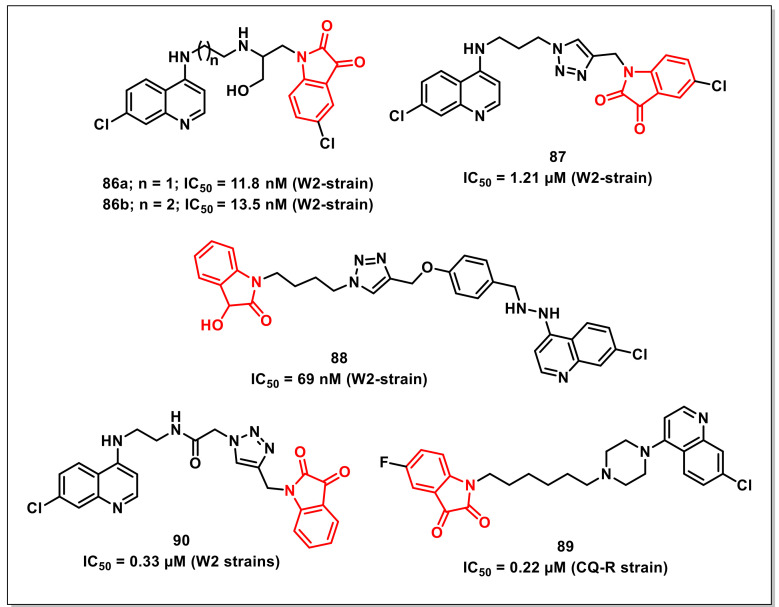
Isatin-7-chloroquinoline hybrids as antiplasmodials.

Nishaet al. synthesized a series of *β*-amino alcohol-linked 4-aminoquinoline-isatin hybrids and evaluated their antiplasmodial activities. The hybrids **86a** and **86b** displayed comparable activities to the antimalarial drug, Chloroquine (CQ), with IC_50_s of 11.8 and 13.5 nM, respectively on a CQ-resistant (W2) strain of *P. falciparum* and were devoid of any cytotoxicity on normal cells (Figure 14) [106].

Rajet al. synthesized a series of 1*H*-1,2,3-triazole-tethered 7-chloroquinoline-isatin hybrids and assessed their activities on a CQ-resistant W2 strain of *P. falciparum*. SAR revealed the dependence of activity on the nature of the substituent on the isatin core as well as the length of alkyl chain introduced as a linker between the two pharmacophores. The C-5 unsubstituted hybrid proved to be the least active among the synthesized series, while the inclusion of chloro substituent improved the antiplasmodial activities. Compound **87**, with an optimum combination of propyl chain as spacer and chloro substituent at the C-5 position of the isatin core proved to be the most potent among the series, exhibiting an IC_50_ of 1.21 μM (Figure 14) [107]. 

Further, the synthesis and antiplasmodial evaluation of 1*H*-1,2,3-triazole-tethered isatin-7-chloroquinoline and 3-hydroxy-indole-7-chloroquinoline hybrids was reported by the same authors. The antiplasmodial activities displayed the dependence of activity on the length of the alkyl chain but independence on the nature of the substituent present at the C-5 position of the isatin or indole ring. The most potent hybrid of the synthesized series, **88**, exhibited an IC_50_ of 69 nM, comparable to that of CQ on the CQ-resistant strain of *P. falciparum* (Figure 14) [108].

Rajet al. further extended the above studies and synthesized a series of 7-chloroquinoline-isatin hybrids either via direct nucleophilic substitution or Cu(I)Cl-promoted Mannich reaction. The newly synthesized hybrids were evaluated for their antiplasmodial activities on a CQ-resistant strain of *P. falciparum*, while cytotoxic profiles were assessed on 3T6 cells. Compound **89** proved to be the most potent of the synthesized series, with an IC_50_ of 0.22 μM and a selective index of 143.73 (Figure 14) [109].

Kumaret al. disclosed the synthesis and antiplasmodial and cytotoxic evaluation of 7-chloroquinoline-based hybrids with isatins/indoles/nitroimidazoles obtained via Cu-promoted click reaction. Among the synthesized series, the hybrids with a shorter alkyl chain length (ethyl/butyl) as spacer displayed better antiplasmodial activities than the ones with longer chain lengths (hexyl/octyl). The most promising hybrid among the synthesized series, **90**, exhibited an IC_50_ of 0.33 μM on the tested strain (Figure 14) [110].

### 4.2. Isatin-Based Schiff’s Bases

**Figure 15:**
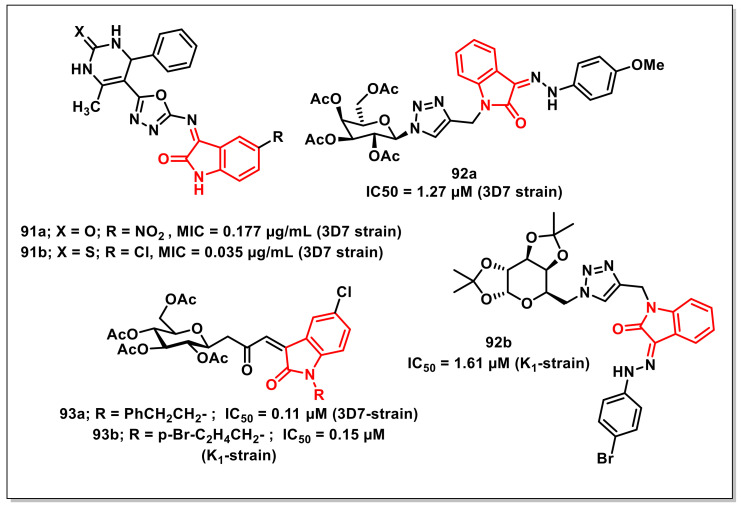
Schiff’s bases of Isatin with antiplasmodial potential.

Akhajaet al. designed, synthesized, and evaluated in vitro tetrahydropyrimidine-isatin hybrids on a CQ-sensitive 3D7 strain of *P. falciparum*. SAR studies revealed that the introduction of a nitro (**91a**, MIC = 0.177 μg/mL) or chloro (**91b**, MIC = 0.035 μg/mL) substituent improved the antiplasmodial activities, with the substitution pattern profiling as NO_2_ > F > Br > H for favourable activity (Figure 15) [111]. 

Thakuret al. synthesized and evaluated a series of glycohybrids of phenylhydrazono-indolinones on CQ-sensitive (3D7) and CQ-resistant (K1) strains of *P. falciparum*. Among the synthesized hybrids, few scaffolds exhibited good antiplasmodial activities, with IC_50_ values < 2 μM on both the tested strains. Compound **92a** proved to be the most active of the series on the 3D7 strain, with an IC_50_ of 1.27 μM, while **92b** was most active on the K1 strain (Figure 15). A SAR study showed that the scaffolds that had acetylated or diacetonide galactose units exhibited better activity on both strains than the ones with acetylated glucose or acetonide xylose units [112]. 

Synthesis of *N*-alkylated 3-glycoconjugated-oxopropylidene oxindoles along with their in vitro antiplasmodial evaluation on both CQ-sensitive and CQ-resistant strains of *P. falciparum* were reported by Thakuret al. Among the synthesized compounds, several exhibited potent activities on the 3D7 strain, with IC_50_ values ranging from 0.1 to 0.3 μM, while some offered promising results on the K1 strain, with IC_50_ values in the 0.1–0.4 μM range, comparable to that of CQ. Compounds **93a** and **93b** proved to be the most active on 3D7 (IC_50_ = 0.11 μM) and K1 (IC_50_ = 0.15 μM) strains, respectively (Figure 15) [113].

### 4.3. Miscellaneous Antiplasmodial Isatin Derivatives

**Figure 16:**
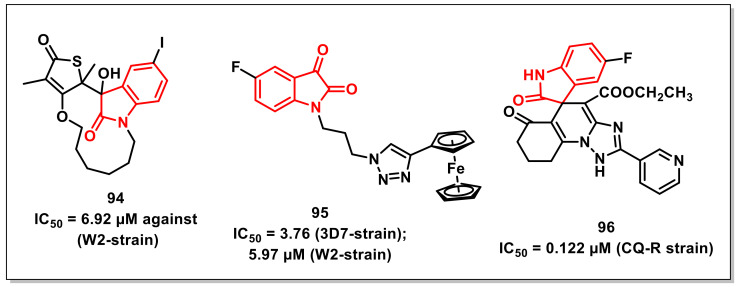
Miscellaneous examples of Isatin-based compounds as antiplasmodials.

A series of thiolactone–isatin hybrids was synthesized by Hanset al. and resulted in the identification of tetracyclic byproducts with superior antiplasmodial potential. The SAR studies ascertained the most potent compound of the series, **94,** with an IC_50_ of 6.92 μM against the CQ-resistant (W2) strain of *P. falciparum* (Figure 16) [114].

Kumaret al. synthesized a series of 1*H*-1,2,3-triazole-tethered isatin-ferrocene hybrids using Cu-promoted click reaction and assayed for their antiplasmodial activities on both 3D7 and W2 strains of *P. falciparum*. Hybrid **95**, along with an optimum combination of halogen substituents at the C-5 position of the isatin ring and a propyl chain as spacer, were the most potent and non-cytotoxic among the series, with IC_50_ values of 3.76 and 5.97 μM against 3D7 and W2 strains, respectively (Figure 16) [115].

Ladaniet al. described an efficient single-pot, three-component reaction of substituted isatin, enaminones, and active methylene, yielding diversely functionalised spiro-indolinone that incorporated 1,2,4-triazolo [1,5-*a*] quinolones, using L-proline as a catalyst. The synthesized compounds were evaluated for their in vitro antibacterial, antiTB, and antiplasmodial activities, respectively. The synthesized scaffolds exhibited IC_50_s in the range of 0.122 to 0.454 μM against *P. falciparum*. The synthesized scaffold, **96**, exhibited an IC_50_ of 0.122 μM, comparable to that of CQ (Figure 16) [116].

## 5. Antimicrobial Activities of Isatin-Based Scaffolds

Antibiotics are unquestionably a blessing to human civilization, having saved millions of lives by combating infections or microbes. Various antibiotics have been used for therapeutic purposes over the years. In the mid-twentieth century, antibiotics were regarded as the “wonder drug”. There was an idealistic belief at the time that communicable disease was on its way out. Antibiotics were thought to be a magic bullet that selectively targeted microbes responsible for disease eradication. Antibiotic resistance progresses rapidly and is therefore a major source of concern. A growing number of infections, such as pneumonia and gonorrhoea, are becoming more difficult and, in some cases, impossible to treat, while antibiotics have become less effective [117].

### 5.1. Isatin-Schiff’s Bases/Oximes

**Figure 17:**
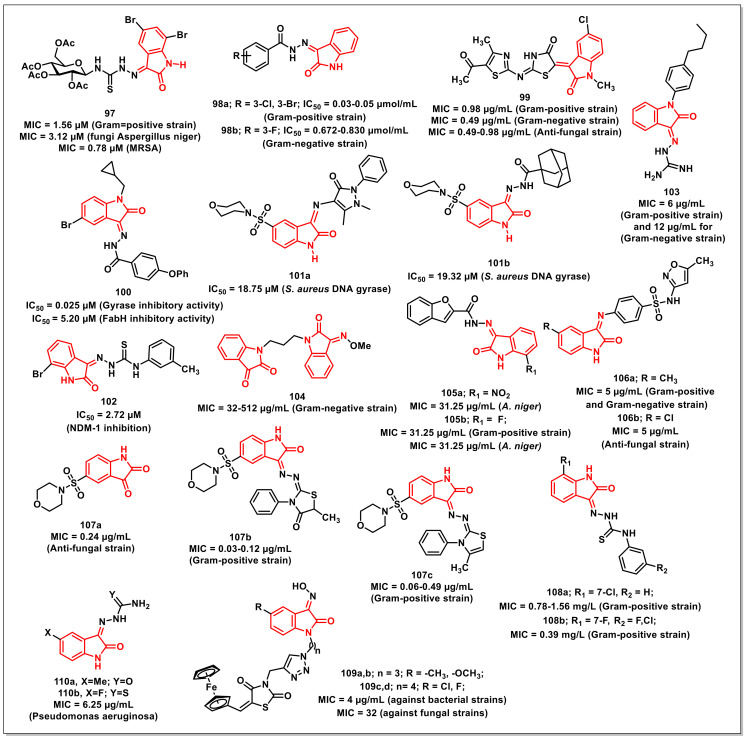
Schiff’s bases and oximes of isatins with antimicrobial potential.

Thanhet al. synthesized a series of isatin-*N*-(2,3,4,6-tetra-*O*-acetyl-β-D-glucopyranosyl) thiosemicarbazones along with an evaluation of their in vivo antioxidant and in vitro antimicrobial activity. The synthesized compounds exhibited MICs of 1.56–6.25 μM and 12.5 μM for Gram-positive and Gram-negative bacteria, respectively. Compound **97**, having bromine at the C-5 and C-7 positions of the isatin ring, proved to be the most active amongst the synthesized thiosemicarbazones, with an MIC of 3.12 μM on *Aspergillus niger* and an MIC of 1.56 μM against *Bacillus subtilis*, *Staphylococcus aureus*, and *Staphylococcus epidermidis* (Figure 17). Additionally, **97** displayed an MIC of 0.78 μM on three clinical MRSA isolates and selective cytotoxic effects on cancer (LU-1, HepG2, MCF7, P338, SW480, KB) and normal fibroblast (NIH/3T3) cells [118].

Lianet al. designed and synthesized isatin derivatives for evaluating their antimicrobial activities. Docking simulations were performed to position these ligands into the FtsZ active site. The synthesized compounds displayed better antibacterial activities against *E. coli*, *P. aeruginosa*, *B. subtilis*, and *S. aureus*. Interestingly, compound **98a** exhibited good antibacterial activities, with IC_50_s of 0.03 and 0.05 μmol/mL against *S. aureus*, respectively (Figure 17). Compound **98b** displayed IC_50_ values of 0.672 and 0.830 μmol/mL on *E. coli* and *P. aeruginosa*, respectively [119].

Abo-Ashouret al. synthesized library of indole-thiazolidinones and evaluated the compounds for in vitro antibacterial and antifungal activities on selected human pathogens viz. *S. aureus*, *P. aeruginosa*, *E. coli*, *M. tuberculosis*, *A. fumigates*, and *C. albicans*. The selective therapeutic index (SI) was determined via eukaryotic cell-toxicity performed through an integrated ex vivo drug screening model. Compound **99** proved to be the most potent among the synthesized series, with broad spectrum antibacterial (MIC: 0.39–0.98 μg/mL); antifungal (MIC: 0.49–0.98 μg/mL), and antiTB activities (Figure 17). Additionally, **99** exhibited potent activity on the resistant bacterial strains methicillin-resistant Staphylococcus aureus (MRSA) and vancomycin-resistant enterococcus (VRE), with an MIC of 3.90 and 7.81 μg/mL, respectively [120]. 

Yanget al. synthesized a series of isatin-Schiff bases aimed at inhibiting the duplication (Gyrase inhibition) and survival (assistant inhibition) activities, simultaneously. The most promising of the synthesized compounds, **100**, exhibited Gyrase inhibitory activity with an IC_50_ of 0.025 μM, while its antibacterial effect was comparable with the reference, Novobiocin (IC_50_ = 0.040 μM) (Figure 17). FabH inhibitory activity (IC_50_ = 5.20 μM) was also successfully presented [121].

Salemet al. synthesized a series of isatin-based Schiff bases and hydrazones and evaluated their antimicrobial activities. Among the synthesized compounds, few of them exhibited significant activities on both Gram-positive and Gram-negative bacteria along with moderate antifungal activities. The evaluation studies of the promising compounds on a multi-drug resistance strain indicated their comparable effectiveness to Norfloxacin and Tetracycline. Compounds **101a** and **101b** proved to be potent inhibitors of *S. aureus* DNA gyrase, with IC_50_ values of 18.75 and 19.32 μM, respectively, which is comparable to that of Ciprofloxacin (26.43 μM) (Figure 17) [122]. 

Songet al. synthesized and evaluated a series of isatin-*β*-thiosemicarbazones for their inhibitory activities on New Delhi metallo-*β*-lactmase-1 (NDM-1). Most of the synthesized compounds proved to be active, with IC_50_s < 10 μM. The most potent scaffold of the series, **102,** with an optimum combination of a bromo-substituent at the C-7 position of the isatin ring and a *m*-tolyl ring, proved to be the most active, with an IC_50_ of 2.72 μM (Figure 17) [123]. 

Wanget al. synthesized a series of isatin-based amphiphilic compounds in order to assess the relationship between molecular hydrophobicity and the antibacterial activity by targeting peptidoglycan glycosyltransferase (PGT). The synthesized compounds were evaluated in vitro for their lipid II transglycosylation inhibitory effects (IC_50_) on *E. coli* PBP1b. Among the synthesized compounds, **103** proved to be most active with an MIC of 6 μg/mL for methicillin-susceptible *Staphylococcus aureus* (MSSA), methicillin-resistant *Staphylococcus aureus* (MRSA), and *B. subtilis* and 12 μg/mL for *E. coli* (Figure 17) [124]. 

Maet al. synthesized and evaluated a series of propylene and butylene-tethered di-isatin heteronuclear hybrids for assessing their antibacterial activities. Most of the synthesized hybrids were active on both Gram-positive and Gram-negative strains, while few of them displayed substantial activities on drug-resistant strains. In particular, compound **104** (MIC = 32–512 μg/mL) proved to be more active than vancomycin on Gram-negative pathogens, while its inhibitory activity was higher than mono-isatin *E. coli* DNA gyrase (Figure 17) [125]. 

Ugaleet al. synthesized a series of isatin-benzofuran-2-carbohydrazides and evaluated their activities. Among the synthesized compounds, **105a,b** displayed excellent antibacterial activity, with MIC = 31.25 μg/mL on *B. subtilis*, *E. coli*, and *P. vulgaris* (Figure 17). Likewise, **105a** and **105b** also exhibited substantial antifungal activity (MIC = 31.25 μg/mL) when compared to fluconazole on *A. niger* [126].

Kandileet al. reported the synthesis of isoxazolyl-isatin-based benzenesulphonamides and explored their in vitro antimicrobial and antifungal activities on a few pathogenic bacterial strains. In vitro evaluation studies revealed compounds **106a** and **106b** to be the most promising of the synthesized series (Figure 17). Compound **106b** (MIC = 5 μg/mL), with the inclusion of chlorine at the C-5 position of the oxindole core, presented more potent antifungal characteristics than **106a** (MIC = 15 μg/mL). Additionally, **106b** demonstrated an MIC = 5 μg/mL against *S. dysenterie* and *B. cereus*, comparable to a standard drug such as sulphamethoxazole [127]. 

Faraget al. synthesized a series of 5-(morpholinosulfonyl)isatins as well as their corresponding hybrids with amino-thiazoles for evaluating their antibacterial activities on a panel of Gram-positive and Gram-negative strains. Among the synthesized scaffolds, 5-(morpholinosulfonyl)isatin (**107a**) showed significant activity against the screened microbes, with MIC values ranging from 0.007 to 0.49 μg/mL (Figure 17). Compound **107a** proved to be four-fold more active than amphotricin B in inhibiting the growth of *A. fumigates* (MIC = 0.24 μg/mL) and displayed two-fold more potency than amphotricin B on *A. clavatus* (MIC = 0.98 μg/mL). Among the thiazole-linked hybrids, compounds **107b** (MIC = 0.03–0.12 μg/mL) and **107c** (MIC = 0.06–0.49 μg/mL) proved to be the most effective on the tested bacterial strains [128].

Zhanget al. reported a series of antibacterial isatin-*β*-thiosemicarbazones with excellent activity against a clinically isolated MRSA (methicillin-resistant Staphylococcus aureus) strain, while few of them exhibited potent inhibition against the VRE (vancomycin-resistant enterococcus) strain. Compound **108a** proved to be the most promising of the synthesized series, exhibiting MICs of 0.78, 1.56, and ≤0.78 mg/L on MRSA, *S. aureus*, and *B. subtilis*, respectively (Figure 17). From an SAR perspective, the presence of a halogen atom at the C-7 position of the isatin ring (R^1^) H on the phenyl ring (R^2^) improved the activities of the synthesized scaffolds. On this rationale, the library was extended, and a series of 41 isatin-*β*-thiosemicarbazones were synthesized. Compound **108b** had an MIC value of 0.39 mg/L against three MRSA strains [129].

The fabrication of a ferrocene-affixed isatin-2,4-thiazolidinedione molecular hybrid connected through a triazole moiety was described by Yagnamet al. A simple copper catalysed alkyne-azide 1,3-dipolar cycloaddition synthesized an isatin-coupled 2,4-thiazolidinedione moiety via a triazole unit. All of the novel entities were screened for antimicrobial efficacy against a variety of ram-positive and Gram-negative bacteria. The compounds **109a**, **109b**, **109c**, and **109d** exhibited MIC values of 4 μg/mL against bacterial strains and 32 μg/mL against fungal strains (Figure 17) [130].

Tehraniet al. synthesized a series of *N*-benzylated isatin-Schiff bases and evaluated their antibacterial activities on a series of Gram-positive and Gram-negative bacterial strains using a microtiter plate method. Among the synthesized series, **110a** and **110b** were the most potent compounds on *Pseudomonas aeruginosa*, with the MIC = 6.25 μg/mL (Figure 17). The SAR analysis revealed that the inclusion of (thio)urea-moiety led to the identification of active compounds with a broader spectrum of antibacterial activity. Additionally, the compounds with high lipophilicity did not exhibit any measurable antibacterial activity, suggestive of the fact that optimal lipophilicity could be significant for the activity of the synthesized compounds [131].

### 5.2. Isatin-Ciprofloxacin/Isatin-Moxifloxacin Hybrids

**Figure 18:**
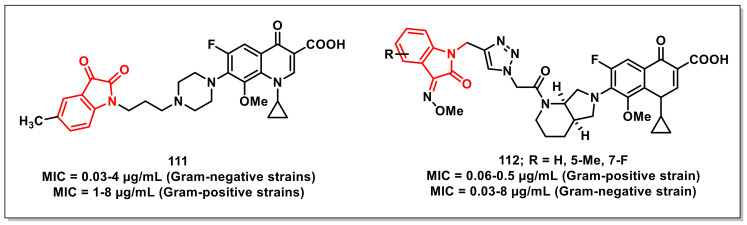
Ciprofloxacin and moxifloxacin-based isatin scaffolds as antimicrobials.

Guoet al. synthesized 8-methoxy-ciprofloxacin (8-OMe CPFX)-isatin hybrids linked via propylene linker and assayed them for their in vitro antibacterial activities on a panel of Gram-positive and Gram-negative pathogens, including drug-resistant bacteria. The synthesized compounds displayed promising antibacterial properties on the tested Gram-negative strains. Among these, **111** proved to be the most active hybrid, with an MIC ranging from ≤0.03 to 8 μg/mL, comparable to the parent 8-OMe CPFX (Figure 18) [132]. 

Gaoet al. synthesized a series of amide-triazole-linked moxifloxacin-isatin hybrids and evaluated for in vitro antibacterial activity on a panel of Gram-positive, Gram-negative bacteria, and drug-resistant pathogens. The synthesized hybrids displayed substantial activity, with an MIC of ≤0.03–128 μg/mL. The non-cytotoxic hybrids **112** displayed comparable activity to that of moxifloxacin (MIC: ≤0.03–8 μg/mL), while the pharmacokinetic profiles of the synthesized hybrids were inferior to moxifloxacin (Figure 18) [133].

### 5.3. Miscellaneous Isatin Scaffolds with Antimicrobial Activities

**Figure 19:**
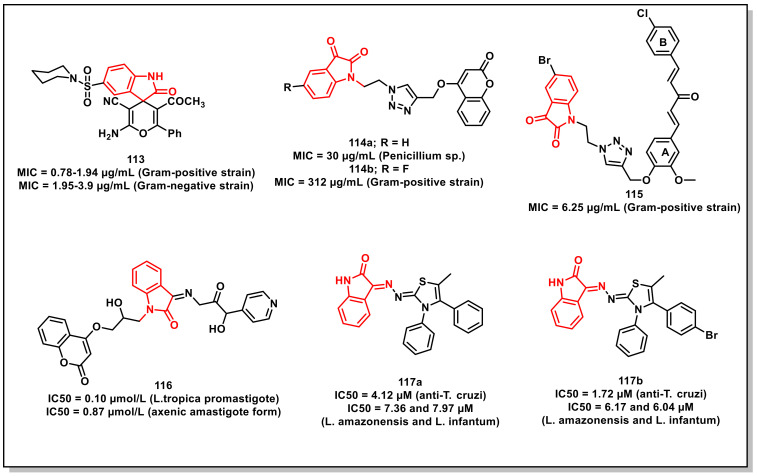
Miscellaneous examples of isatin-based compounds as antimicrobials.

Salemet al. synthesized a series of 2-oxospiro[indoline-3,4’-pyran] derivatives via the single pot reaction of substituted indole-2,3-diones, appropriate nitriles, and *β*-dicarbonyl compounds, and evaluated for their in vitro antibacterial, antifungal, and immunomodulatory activity. Most of the synthesized compounds displayed high activity in killing pathogens, with a good MBC value against norfloxacin, and were also investigated on an extended panel of MDR bacteria. The most potent compound of the series, **113**, displayed an increase in the intracellular-killing activity of neutrophils (Figure 19). It showed that MIC = 0.78 μg/mL against *S. aureus* ATCC 33,591 and MIC = 1.95 μg/mL against *P. aeruginosa* ATCC BAA-2111 [134].

Bhagatet al. synthesized and evaluated a series of indolindione-coumarin hybrids for their antimicrobial activities on Gram-negative (*E. coli* and *S. enterica*), Gram-positive (*S. aureus* and *M. smegmatis*), and fungal strains (*C. albicans*, *A. mali*, *Penicillium* sp., and *F. oxysporum*). The compounds **114a** and **114b** proved to be most promising, with MIC 30 and 312 μg/mL for *Penicillium* sp. and *S. aureus*, respectively (Figure 19). SAR studies revealed that the electronic nature of substituents on the isatin core as well as the length of the alkyl chain between the two pharmacophores substantially affected the antimicrobial properties of the synthesized hybrids. Molecular docking studies of **114b** in the active site of *S. aureus* dihydrofolate reductase confirmed Van der Waal’s π–π stacking and H-bonding interactions and deciphered its mechanism of action [135]. 

Singhet al. synthesized and evaluated a series of 1*H*-1,2,3-triazole-tethered curcumin-coumarin and curcumin-isatin hybrids for their antibacterial activities on Gram-positive (*E. faecalis* and *S. aureus*) and Gram-negative (*P. aeruginosa* and *E. coli*) bacterial strains. Among the synthesized hybrids, **115** proved to be the most promising, with an MIC of 6.25 μg/mL (Figure 19). SAR studies revealed the presence of an ethyl chain as a spacer along with the bromo substituent at the C-5 position of the isatin ring, –chloro at the C-4 position of ring-A, and –OCH_3_ at C-2 on ring-B of curcumin to be optimum for antibacterial activity. Molecular modelling studies of **115** were also carried out in the active site of DHFR so as to study the various modes of binding interactions [136]. 

Khatoonet al. reported the in silico and in vitro evaluations and synthesis of coumarin-incorporated isatin hydrazones as antileishmanial agents. Molecular docking was first used to determine the binding confirmation of lead molecules to the target protein. Only three of the docked compounds demonstrated high-binding affinities. These compounds were then tested for antileishmanial activity against *Leishmania tropica* promastigotes and amastigotes and were found to be active, with IC_50_ values ranging from 0.1 to 4.13 μmol/L. Compound **116** was found to be the most effective, with IC_50_ values of 0.10 and 0.87 μmol/L against *L. tropica* promastigote and axenic amastigote forms, respectively (Figure 19) [137].

Freitaset al. synthesized thiazolyl-isatin derivatives from thiosemicarbazone or phenyl-thiosemicarbazone and tested their activity against Trypanosoma cruzi. Compounds **117a** (IC_50_ = 4.12 μM) and **117b** (1.72 μM) exhibited the best anti-*T. cruzi* activity for the trypomastigote form, with a selectivity index higher than benznidazole (BZN) (Figure 19). SEM analysis revealed that *T. cruzi* trypomastigote cells treated with compound **117b** induced changes in the shape, flagella, and surface of the body, resulting in parasite death. Compounds **117a** (IC_50_ = 7.36 and 7.97 μM, respectively) and **117b** (6.17 and 6.04 μM, respectively) demonstrated the best activity for the promastigote form among the series, as well as a higher selectivity than Miltefosine [138]. 

## 6. Conclusions

Isatin is a highly promising scaffold in drug discovery due to its ubiquitous presence in biological systems; it is a molecular architecture that can be easily modulated in addition to a plethora of biological activities. Of course, isatin’s anticancer potential is overwhelming, as evidenced by the number of isatin-based compounds in use as therapeutics or in various stages of clinical trials. Isatin derivatives have demonstrated promising antiproliferative attributes against various cancer cells, targeting specific biomolecules or organelles as free ligands or those coordinated to metal ions. Adherence to metal ions frequently enhances its bioactivities, indicating a synergistic mechanism comprising the metal and the ligand. They also disclose a variety of modes of action, including the ability to bind DNA, generate reactive species that induce oxidative damage, and suppress specific proteins. Isatin-pharmacophore hybrids have the potential to overcome drug resistance and provide new functional entities with multiple mechanisms of action and good safety profiles. In the case of antimycobacterials, the inclusion of isatin with isoniazid has not only improved the lipophilicity as well as the activity of the hybrids, but also minimized the frequency of the development of resistance. The inclusion of isatin core with quinoline core afforded hybrids better antiplasmodial profiles than CQ itself against the CQ-resistant species of *P. falciparum*. The current evidence therefore suggests that further exploiting this promising moiety can provide efficient clinical candidates with a reduced incidence of drug resistance. We anticipate that introducing isatin into various drugs/organic moieties will significantly impact the treatment of various diseases in the near future, given the multitude of biological activities and mechanistic pathways provided by isatin-based compounds.

## Data Availability

Data sharing not applicable.

## Abbreviations

NTDS, Neglected Tropical Diseases; WHO, World Health Organization; DNDi, Drugs for Neglected Diseases Initiative; CNS, central nervous system; ATP, adenosine triphosphate; nM, nanomolar; μM, micromolar; DNAP, 3,5-diaryl-*N*-acetyl-pyrazolines; 5-FU, 5-fluorouracil; SRB, sulforhodamine B; FDA, Food and Drug Administration; DNA, Deoxyribonucleic acid; BSA, Bovine serum albumin; IC_50_, Half-maximal inhibitory concentration; GI_50_, half maximaL growth inhibition; EC_50_, half maximal effective concentration; TB, Tuberculosis; *Mtb*, Mycobacterium tuberculosis; MDR, Multidrug-resistant; HIV, human immunodeficiency virus; CPFX, Ciprofloxacin; SAR, Structure-Activity Relationship; MXFX, moxifloxacin; INH, isonicotinic acid hydrazide; CC_50_, half maximal cytotoxity concentration; MIC, Minimum inhibitory concentrations; NPs, nanopartocles; PAS, para-amino salicylic acid; 3D-QSAR, 3-Dimentional Quantitative Structure-Activity Relationship; INH-R, isoniazid-resistant; RIF-R, rifampicin-resistant strains; FQ-R, fluoroquinolone-resistant; CQ, Chloroquine; MRSA, methicillin-resistant Staphylococcus aureus; VRE, vancomycin-resistant enterococci; MSSA, methicillin-susceptible *Staphylococcus aureus*; NDM-1, New Delhi metallo-*β*-lactmase-1; BZN, benznidazole.
